# Human subsistence and signatures of selection on chemosensory genes

**DOI:** 10.1038/s42003-023-05047-y

**Published:** 2023-07-03

**Authors:** Carrie C. Veilleux, Eva C. Garrett, Petar Pajic, Marie Saitou, Joseph Ochieng, Lilia D. Dagsaan, Nathaniel J. Dominy, George H. Perry, Omer Gokcumen, Amanda D. Melin

**Affiliations:** 1 Department of Anatomy, Midwestern University, 19555 N 59th Ave, Glendale, AZ 85308 USA; 2 Department of Anthropology & Archaeology, University of Calgary, 2500 University Drive NW, Calgary, AB T2N 1N4 Canada; 3 Department of Anthropology, Boston University, 232 Bay State Road, Boston, MA 02215 USA; 4 Department of Biological Sciences, University at Buffalo, 109 Cooke Hall, Buffalo, NY 14260 USA; 5 Department of Anatomy, Makerere University College of Health Sciences, Kampala, Uganda; 6 National Commission for Indigenous Peoples, Botolan, Philippines; 7 Department of Anthropology, Dartmouth College, 6047 Silsby Hall, Hanover, NH 03755 USA; 8 Departments of Anthropology and Biology, The Pennsylvania State University, 410 Carpenter Building, University Park, PA 16802 USA; 9 Department of Medical Genetics, University of Calgary, 3330 Hospital Drive NW, Calgary, AB T2N 4N1 Canada; 10 Alberta Children’s Hospital Research Institute, 3330 Hospital Dr. NW, Calgary, AB T2N 4N1 Canada

**Keywords:** Olfactory receptors, Taste receptors, Biological anthropology, Agriculture, Molecular ecology

## Abstract

Chemosensation (olfaction, taste) is essential for detecting and assessing foods, such that dietary shifts elicit evolutionary changes in vertebrate chemosensory genes. The transition from hunting and gathering to agriculture dramatically altered how humans acquire food. Recent genetic and linguistic studies suggest agriculture may have precipitated olfactory degeneration. Here, we explore the effects of subsistence behaviors on olfactory (*OR*) and taste (*TASR*) receptor genes among rainforest foragers and neighboring agriculturalists in Africa and Southeast Asia. We analyze 378 functional *OR* and 26 functional *TASR* genes in 133 individuals across populations in Uganda (Twa, Sua, BaKiga) and the Philippines (Agta, Mamanwa, Manobo) with differing subsistence histories. We find no evidence of relaxed selection on chemosensory genes in agricultural populations. However, we identify subsistence-related signatures of local adaptation on chemosensory genes within each geographic region. Our results highlight the importance of culture, subsistence economy, and drift in human chemosensory perception.

## Abstract (web summary)

Genetic comparison of rainforest foraging and neighboring agricultural communities in Uganda and the Philippines shows no distinction in the size of olfactory receptor gene repertoires, but there is evidence for subsistence-related local adaptation.

## Introduction

Transitions from hunting and gathering to agricultural food production were formative events during human history, beginning 11.5 kya and occurring independently in multiple geographic regions^1,2^. Agricultural foodways have fueled profound changes––to human environments, behavior, health, and social structure^3–5^, but also dietary ecology, resulting in diminished food diversity and shifts in the types and proportions of nutrients consumed^5–7^. Mounting evidence from studies of modern and ancient human populations point to attendant selective pressures on the sequences and diversity of genes underlying digestion, metabolism and growth^8–13^, sometimes convergently between agricultural origin centers^7,8,10,11^. But the effects of agriculturalization on the human sensorium are understudied despite the essential role of chemosensation (olfaction, taste) during food detection and assessment (e.g., identifying fruit ripeness, the presence of toxins, or spoilage)^7,14–20^.

Shifts in feeding behavior appear to drive changes in the olfactory receptor (*OR*) and taste receptor (*TASR*) genes of some species^21–25^, which raises the possibility of similar differentiation between human populations. Transitions from forest-foraging to agriculturalism are expected to exert selective pressure on human chemosensory genes for at least two reasons. First, farming clears land in a way that reduces humidity and increases temperatures at ground-level, which increases convective air turbulence and hinders chemotaxis (detection and movement toward odorants)^26,27^. Second, agriculturalists are more sedentary and reliant on a subset of available foods, mostly domesticates^6^, which limits their exposure to available chemosensory stimuli in the natural environment. Farming may therefore accompany a relaxation of natural selection, leading to impoverished chemosensory gene repertoires. Conversely, rainforest foragers are expected to retain greater numbers of functional *OR* genes for enhanced odor detection and discrimination^16,28,29^. Exemplifying this pattern are language studies––the diversity of olfactory words and expressions is striking in the languages of some rainforest foragers^30^, as are their abilities to name odors relative to nearby agriculturalists speaking closely-related languages^31,32^. Taste sensitivities can also differ between rainforest foragers and neighboring farmers^33^, and there is growing evidence of diet-driven haplotype variability at local scales^8,10,11,34^. Yet when comparative studies of *TASR* genes have included foraging and farming peoples, the focus has been limited to single genes or a few gene regions^15,29,35–37^, rather than a wider genomic-scale approach. Further, there was little effort to compare adjacent populations of foragers and farmers.

**Fig. 1:**
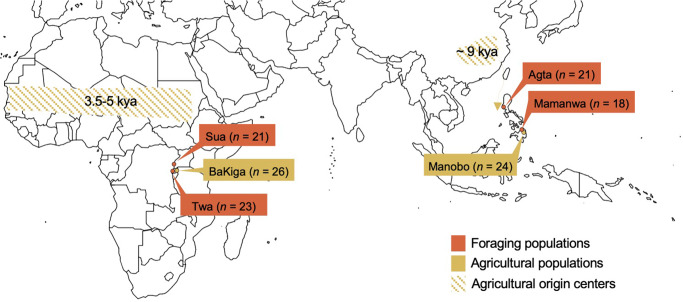
Rainforest traditional foraging and agricultural populations collaborating in this study and sample sizes. Shaded regions reflect estimated agricultural origin center locations and dates^1,2^. Map adapted from Fla-Shop (www.fla-shop.com) and licensed by Creative Commons (CC-BY-4.0).

We have conducted recurring community-based participatory research since 2008. Our work in Uganda and the Philippines has been focused on Indigenous knowledge systems, belonging, and restorative justice among neighboring communities with different subsistence economies and histories (Fig. 1). This background motivated us to sequence the chemosensory gene repertoires (378 *OR* genes, 2 *TAS1R* genes, 24 *TAS2R* genes) of 133 individuals across six populations. Four of the populations – the Agta, Mamanwa, Sua, and Twa – turned increasingly, but at different moments and to varying degrees, toward agricultural foodways during the last century (Supplementary Note 1). We will refer to these populations as ‘traditional foragers’ to distinguish them from the long-term agriculturalists in our study, the BaKiga and Manobo (Supplementary Note 1). Although the word ‘traditional’ risks accusations of essentialism—or freezing people in “a simulacrum of pastness,” as Rifkin^38^ put it—it best represents the lived experiences or collective memories of most study participants. This geographic-neighbors study design in Africa and Asia has two advantages. It accounts for higher levels of genetic diversity within Africans relative to non-Africans^29,39^ and provides independent tests on the effects of parallel transitions toward agriculturalism.

Here we ask two complementary questions. First, does agriculturalism relax the selective constraints acting on chemosensory genes as evidenced by relatively higher frequencies of pseudogenes? And second, do signatures of positive selection or local adaptation exist as a function of subsistence history in one or both geographic regions, as determined using cross-population population differentiation based tests? Our overarching objective is to improve our understanding of human sensory evolution and perception.

## Results

### Is agriculturalism associated with a relaxation of selective constraint on chemosensory genes?

**Fig. 2:**
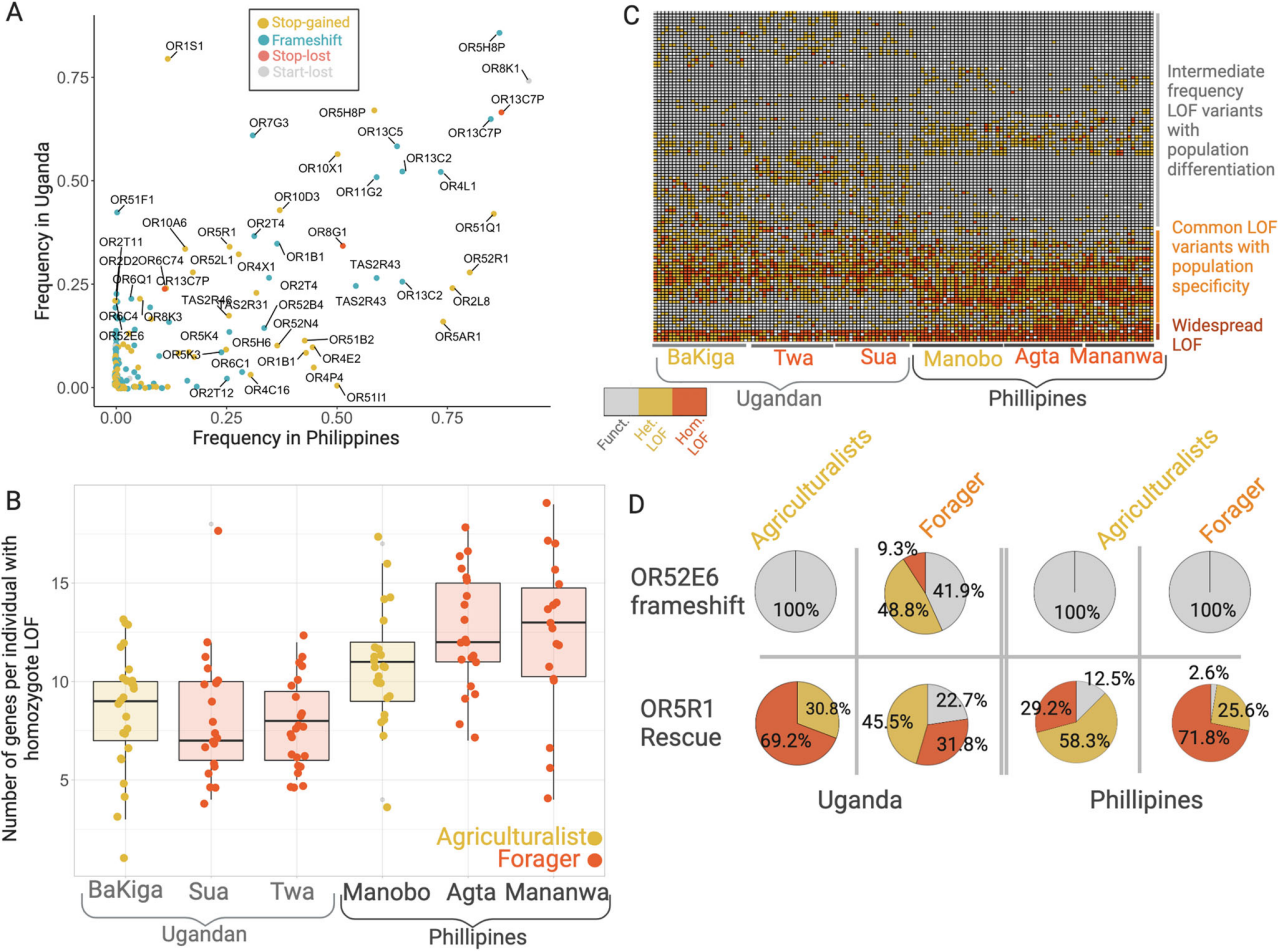
The distribution of loss-of-function (LOF) variants. **A** The frequency of LOF variants in populations in Uganda and in the Philippines. **B** Boxplot of the distribution of the number of genes carrying homozygous LOF variants in each individual. **C** Heatmap of LOF variants in each sample genotyped in this study color-coded as functional variant (gray), heterozygous LOF variant (gold), or homozygous LOF variant (orange). Only variants with allele frequency higher than 5% in Ugandan or Philippine population are shown. **D** The allele frequencies of two LOF variants that show the highest population difference between agriculturalists and traditional foragers.

We employed two approaches to investigate the impact of subsistence strategy on the selective constraint acting on chemosensory gene repertoires. First, we examined patterns of gene loss-of-function (LOF) mutations. We identified 235 LOF variants (SNPs, indels) across 161 *OR* genes and 17 LOF variants across 12 *TASR* loci (Supplementary Data S1). We found that 76 (30%) of the LOF variants are shared between populations from Uganda and the Philippines, however, 4.7 times more LOF variants are specific to the populations from Uganda (*n* = 142) than specific to populations in the Philippines (*n* = 33) (Fig. 2A). In contrast to the higher number of variants, we found that on average, individuals from Asia have a higher number of homozygous, putatively non-functioning, chemosensory genes (11.78) than individuals from Africa (8.22) (one-tailed Wilcoxon Test, W = 3505, *p* < 0.000001) (Fig. 2B, C). It is important to note that our analysis assumes a model of recessive deleterious alleles, such that heterozygotes are not considered to have lost gene function, and that we likely underestimated the number of homozygous LOF genotypes due to the relatively high level of false-negatives in our variant calling pipeline. However, our results should be unbiased to any particular population/subsistence history and hence provide a global overlook to the diversity of perception genes across the globe.

Under relaxed selection, we would expect a higher number of homozygous LOF variants, on average, in populations with a deeper history of agriculturalism compared to traditional foraging in each geographic region. Overall, however, the number of functional genes did not show clear and consistent differentiation between the agriculturalists and traditional foragers (Fig. 2C). Specifically, in Africa, individuals from the agricultural and traditional foraging populations carry on average 8.02 and 8.58 homozygous LOF variants, respectively, with no significant difference (one-tailed Wilcoxon Test, W = 670, *p* = 0.233). We similarly found no significant difference when separately comparing the number of homozygous LOF variants between BaKiga and the Sua (W = 306, *p* = 0.483) or the BaKiga and the Twa (W = 364, *p* = 0.193). In Asia, individuals from the agricultural and traditional foraging populations did significantly differ in the number of homozygous LOF variants (W = 313.5, *p* = 0.028), with the traditional foraging populations carrying on average 12.43 homozygous LOF variants versus the agriculturalist’s 10.71 homozygous LOF variants. When compared separately, neither the Agta (W = 334, *p* = 0.062) nor the Mamanwa (W = 288.5, *p* = 0.066) significantly differed in the number of homozygous LOF variants relative to the Manobo. However, the difference in homozygous LOF load between continental African and Asian populations (Fig. 2B) is nearly twice that between Philippine foragers and agriculturalists. Subsistence history was also not significant in explaining the number of homozygous LOF genes carried per individual using a linear mixed effects model (*p* = 0.259), which controls for variation in the number of genes called per individual, population, and continent of origin. While some segregating LOF variants do show differences between agriculturalist and traditional foraging populations within continents (Fig. 2D, Supplementary Note 2), the majority of variance in the distribution of homozygous LOF variants is shaped by continental ancestry rather than subsistence history.

**Fig. 3:**
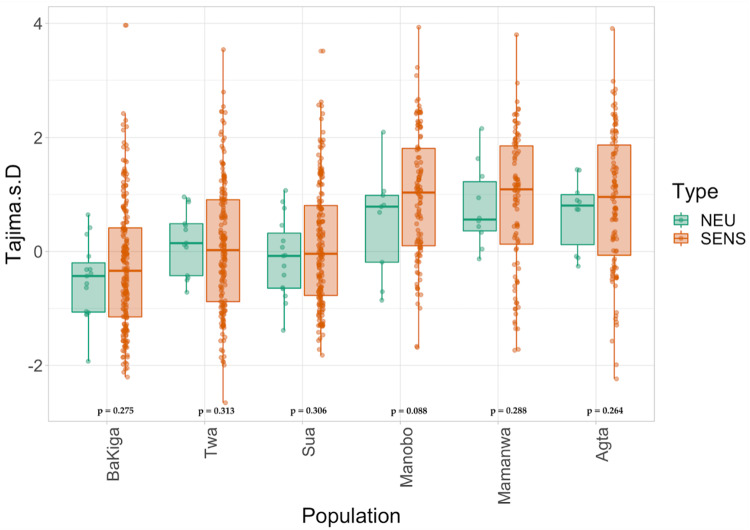
Boxplot of Tajima’s D values for each interval (1000 bp) containing chemosensory genes (orange, SENS) and for chromosomes putatively “neutral” SNPs (green, NEU). We calculated genome-wide Tajima’s D values to construct a neutral distribution for each chromosome where we have more than 3 neutral SNPs, representing chromosome-level estimates of Tajima’s D under neutrality. In contrast, for each chemosensory gene for which we have 3 or more SNPs, we calculated Tajima’s D for 1000 bp intervals. Boxplots depict first to third quartiles with median at center line and whiskers as minimum/maximum excluding outliers. Points reflect raw data. P-values reflect the results of the one-tailed Wilcoxon rank sum tests comparing Tajima’s D values between chemosensory and neutral SNPs.

For our second approach, we calculated Tajima’s D values^40^ for each population to directly test the hypothesis of differing levels of relaxed versus purifying selection associated with subsistence history (Fig. 3). Negative Tajima’s D values indicate purifying selection. However, this measure is affected by demographic history as well. Thus, we tested whether Tajima’s D values for chemosensory intervals show significantly lower values than neutral intervals for traditional foragers, but not agriculturalists. We found no significant differences in Tajima’s D between the chemosensory and putatively neutral intervals for any population (Fig. 3), suggesting that similar selective forces are acting on the chemoperception genes across populations. Hence, the observed differences in the distribution of Tajima’s D values between populations in the Philippines and Uganda are likely due to recent population expansion and bottleneck events, respectively. Together, the results of LOF and Tajima’s D analyses suggest that there are no genome-wide trends of differential selection acting on *OR* or *TASR* genes between populations due to subsistence history. To understand overall genetic variation shaped by demographic history, we also calculated genome-wide nucleotide variation (**π**) (Supplementary Fig. S1). We observed, as expected, similar **π** values among populations from the same geography (Uganda vs. Philippines). We further found that Ugandan populations have higher nucleotide diversity independent of their subsistence histories. These results further support the interpretation that the variation we observed in these populations are likely a product of different demographic histories, rather than population-specific adaptation.

### Has subsistence strategy influenced positive selection on chemosensory genes?

**Fig. 4:**
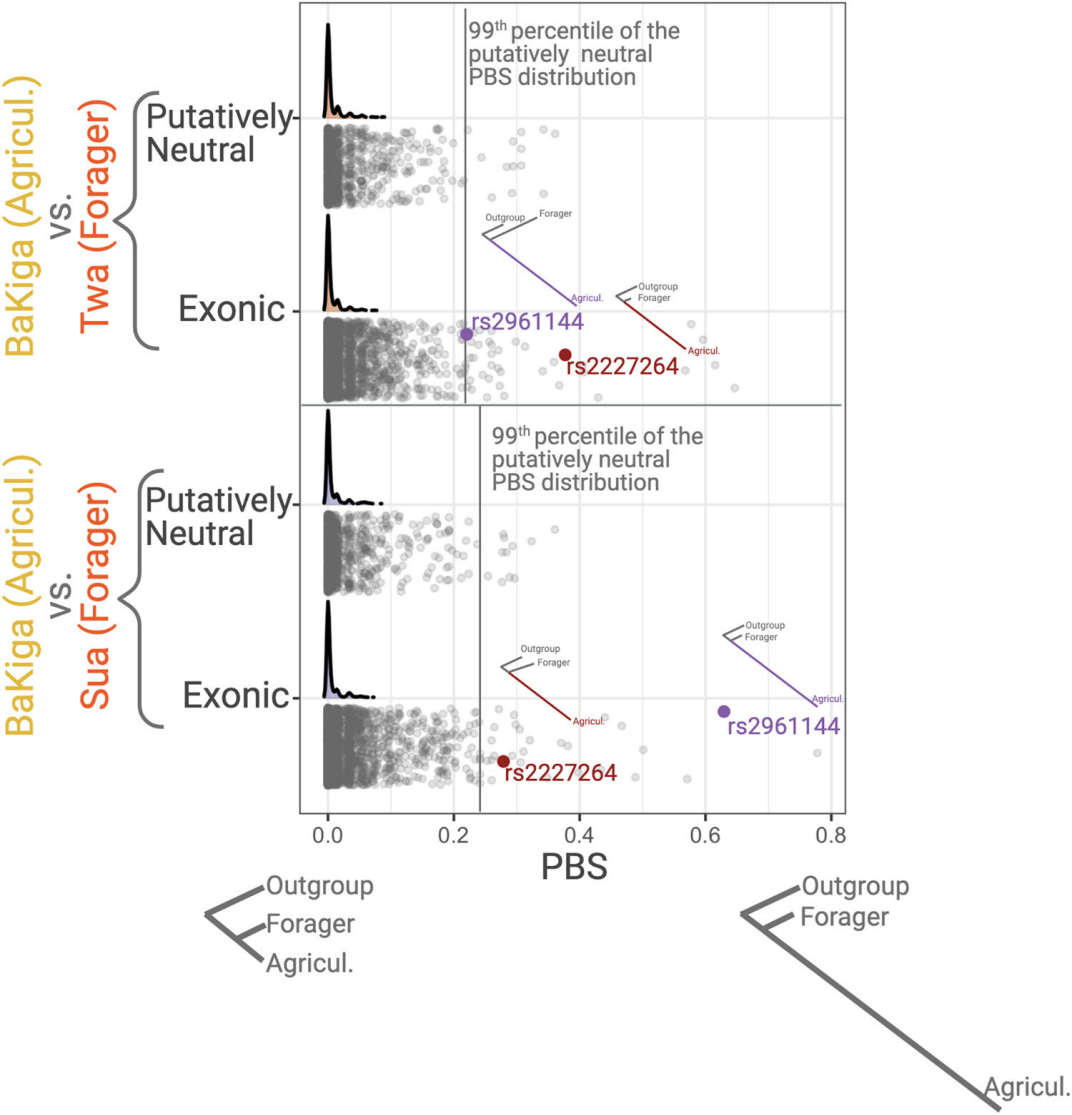
An example of the distribution of PBS-values for putatively neutral and putatively functional variants for two focal population pairs investigating selection in the agricultural population. The top and middle panels show the comparison of BaKiga with Twa and Sua, respectively. The SNPs that we focused on for investigating the functional impact of putatively adaptive haplotypes are highlighted (rs2227264 in *TAS2R5*, rs2961144 in *OR2A5*). The trees on the bottom panel indicate the concept of the PBS test, with the left tree depicting putatively neutral expectations and the right tree expectations of a SNP under positive selection in the agricultural population. Note the trees for rs2227264 and rs2961144 in the top and middle panels.

We performed population branch statistics (PBS)^41^ to search for signatures of recent positive selection on chemosensory genes, identifying SNPs that show unusually high allele frequency in the agriculturalist populations relative to the traditional foraging populations as well as those SNPs that show unusually high allele frequencies in the traditional foraging populations. We used empirical tests to detect chemosensory SNPs that significantly diverged from neutral expectations (Fig. 4). After correcting for multiple testing using the false discovery rate, we detected 17 SNPs in 8 genes that significantly differed from neutral expectations in the Ugandan agriculturalist population and 8 SNPs in 3 genes that differed in the traditional foraging populations in Uganda (Supplementary Table S1). In Philippine populations, 2 SNPs in 2 genes and 16 SNPs in 13 genes significantly differed from neutral expectations in the agricultural and traditional foraging populations, respectively (Supplementary Table S2). Many of these genes (15 out of 26) have been identified in previous positive and/or local selection scans in humans (Supplementary Tables S1 and S2). Furthermore, several have been linked to specific phenotypic effects in previous studies, including agonizts, food preferences, food intake, or obesity (Supplementary Tables S1 and S2). We next conducted a PheWAS search to explore significant trait associations for these putatively adaptive SNPs using the UK Biobank Gene Atlas and the GWAS Atlas. We identified significant trait associations for 35 of the 43 total SNPs, including particularly relevant traits such as “processed meat intake”, “coffee/tea intake”, “fresh fruit intake”, and “bread intake” (Supplementary Tables S3 and S4). Additionally, 33 SNPs are physically close (within 1 Mb of each other) and likely represent ten haplotype blocks (Supplementary Table S5). We note that these haplotype blocks are likely incomplete as our capture method only detects a subset of mostly exonic SNPs. Hence, the lengths of the haplotypes should be extrapolated using other, more comprehensive datasets.

We targeted six of these putative haplotype blocks with clear phenotype associations for further investigation (Table 1). Using 5008 haplotypes from the 1000 Genomes Project data, we first confirmed the high linkage disequilibrium of the SNPs with high PBS values in our dataset to each other (R^2^ > 0.7).We next explored the evolutionary history of the SNPs in these haploblocks using ages available through Human Genome Dating database (Table 1). The target SNP in 5 of these 6 haploblocks is relatively ancient (~619 Kyr to 1.47 Myr), predating the origin of anatomically modern humans. Only one SNP and haploblock (rs12360890, *OR5T1*) evolved relatively recently (126 Kyr). While *OR5T1* and this specific SNP are associated with age at menarche, other genes in its associated haploblock include tropical adaptation in cattle and a honey/floral odorant (Supplementary Table S5).

**Table 1:** Allele frequencies and estimated age (Est. Age) of select haplotypes from PBS and PheWAS analyses.

Position	Linked genes	PheWAS or other association	SNP	Ref	Alt	Anc	Eff	Est. age	Agta AF	Mamanwa AF	Manobo AF	Sua AF	Twa AF	BaKiga AF	Asia AF	Africa AF
Chr7: 141490238	*TAS2R3*^*UA*^ *TAS2R4* ^*UA*^ *TAS2R5* ^*UA*^	Salt added to food; Bitterness of ethanol, capsaicin, espresso	rs2227264^m^	G	T	T	G	618.6 Kyr	0.73	0.50	0.59	0.39	0.48	0.17	0.61	0.34
Chr7: 143747870	*OR2A5* ^*UA*^	Fresh fruit/ Bread intake (A)	rs2961144^m^	A	G	A	G	725.6 Kyr	0.05	0.00	0.10	0.15	0.45	0.65	0.06	0.44
Chr9: 125330739	*OR1L8* ^*UA*^	Coffee: instant	rs1999182^S^	G	A	A	G	1.47 Myr	1.00	1.00	1.00	0.63	0.33	0.30	1.00	0.41
Chr11: 56043604	*OR5T1*^*PF*^ *OR5R1* ^*PF*^ *OR8H1* ^*PF*^ *OR8K1* ^*PF*^	Menarche; tropical adaptation in cattle, MCMP odorant	rs12360890^m^	A	G	N'	A	126 Kyr	0.81	0.81	0.48	0.02	0.02	0.19	0.68	0.09
Chr12: 11338781	*TAS2R42* ^*UF*^	Coffee/Tea intake(A)	rs1669413^m^	C	A	A	C	901.7 Kyr	0.98	1.00	1.00	0.17	0.37	0.48	0.99	0.35
Chr19: 9237542	*OR7G3* ^*PF*^	Processed Meat/impedance, eating behavior, BMI, body fat	rs10414255^m^	T	C	T	C	489.1 Kyr	0.35	0.07	0.45	0.61	0.56	0.77	0.32	0.66

For Linked Genes, superscript indicates whether genes with outlier SNPs were derived in the Ugandan agriculturalists (UA), Ugandan foragers (UF), or Philippine foragers (PF). For SNPs, superscript indicates functional consequence: synonymous (s) or missense (m). ‘Ancestral allele is unknown (N). Abbreviations: reference allele (Ref), alternate allele (Alt), ancestral allele (Anc), effect allele (Eff), estimated age (Est. Age), and frequency of the alternate (non-reference) allele (AF). “Asia” is the average allele frequency of the Philippine populations, “Africa” is the average allele frequency of the Ugandan populations in this study. For Associations, the allele for a given PheWAS haplotype association is assumed to be the effect allele unless otherwise indicated in parentheses. References for associations are in Supplementary Tables S1–S4.

**Fig. 5:**
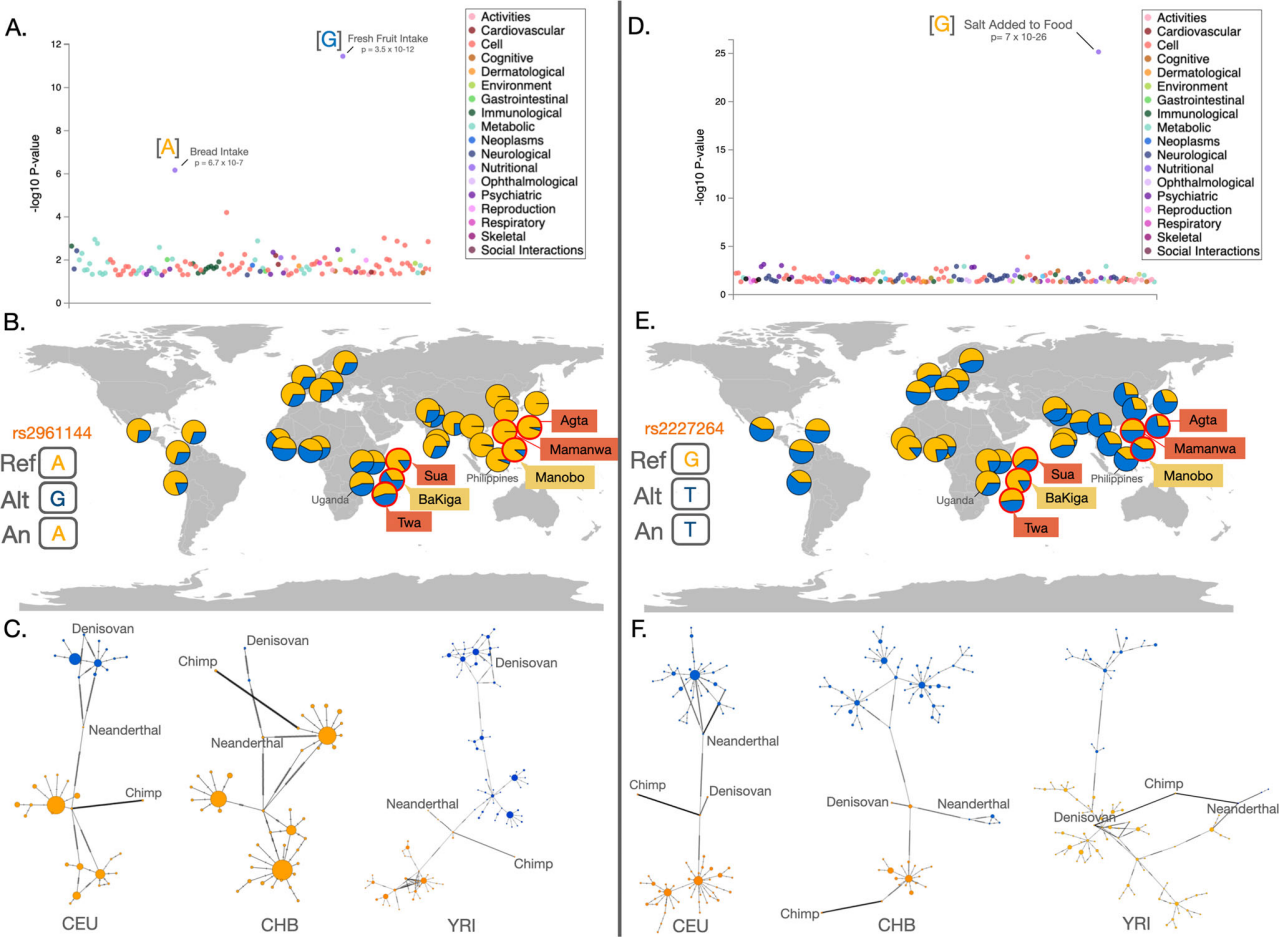
SNP PheWAS and allele frequencies of OR2A5 and TAS2R3/4/5 haplotypes with phenotypic associations to fruit and bread intake and to bitterness perception of ethanol and spices, respectively. **A**, **D** PheWAS analysis with associated allele(s) in brackets. **B**, **E** The allele frequency distribution of the rs2961144 and rs2227264 SNPs, respectively. Frequency pie charts represent major 1000 Genomes haplotype groups across global populations (Africans: [ACB, ASW, ESN, GWD, LWK, MSL, YRI], Asians: [BEB, CDX, CHB, CHS, GIH, ITU, JPT, KHV, PJL, STU], Europeans: [CEU, FIN, GBR, IBS, TSI]), with red circles highlighting the traditional foragers (Sua, Twa, Agta, and Mamanwa) and agriculturalists (BaKiga, Manobo) in this study. The gold color indicates the frequency of the haplotypes in each population carrying the reference allele, whereas blue are haplotypes representing alternative alleles. **C**, **F** Phylogenetic haplotype network analysis for each SNP on 3 human populations: Central European (CEU), Han Chinese (CHB) and Yoruba (YRI), and including Neanderthal, Denisovan, and chimpanzee. Map generated in *rworldmap* R package^95^.

Two haploblocks are of particular interest in regards to subsistence strategy given their functional associations in humans (Fig. 5: *OR2A5* and *TAS2R3/4/5*). Both are relatively ancient (727 Kyr and 619 Kyr, respectively). At the *ORA25* haploblock (chromosome 7), the ancestral haplotype is associated with a preference for “bread intake” and the derived haplotype associated with a preference for “fruit intake” (Fig. 5A, Supplementary Table S3). The “fruit intake” haplotype is at a much higher frequency in the Ugandan BaKiga agriculturalist population relative to the neighboring Sua and Twa, who were committed hunter-gatherers before the 1990s (Fig. 5B). The *TAS2R3/4/5* haploblock is also found on chromosome 7, and is associated with bitterness perception of ethanol and capsaicin^42^, alcohol consumption, and “adding salt to food” (Fig. 5D, Supplementary Table S5). For three of four neighboring pairs of agriculturalist/foraging populations in our study, the agriculturalists exhibited a higher frequency of the haplotype associated with decreased bitterness perception of ethanol and capsaicin/piperine, increased alcohol consumption, and adding salt to food, while the traditional foraging populations have a higher frequency of the haplotype associated with increased bitter perception of those compounds (Fig. 5E, Table 1). We describe the variation and evolutionary history of both the *OR2A5* and *TAS2R3/4/5* haploblocks in more detail in the Supplementary Note 3.

Overall, the results of our PBS analyses provide a list of candidate haplotypes and SNPs in *OR* and *TASR g*enes with strong allele frequency differentiation between neighboring populations with different subsistence histories. These may be worthwhile candidates for future investigation.

## Discussion

In this study, building on our collaborative relationships with foraging peoples in Uganda and the Philippines, we used a geographic-neighbors population genomic approach to investigate how subsistence history has influenced the evolution of chemosensory genes. We sequenced the chemosensory gene repertoire (378 *OR* genes, 2 *TAS1R* genes, and 24 *TAS2R* genes) of 133 individuals across six populations. Overall, we do not find strong evidence in support of previous claims that foraging peoples possess more diverse olfactory receptor gene repertoires. However, we do find evidence of local adaptation via differences in allele frequencies with implications for associated phenotypes. We conclude by highlighting potentially fruitful future directions.

We identified 252 loss-of-function (LOF) variants (SNPS, indels) in the chemosensory gene repertoires across six populations (235 in *OR* genes, 17 in *TASR* genes). Previously, researchers have suggested that hunter-gatherers have a higher number of functioning *OR* genes relative to agriculturalists, which was attributed to higher reliance on olfaction for finding and assessing foods in the former^28,29,36^. We found no evidence of this in our study. Among the Ugandan populations, there was no significant difference in the number of homozygous LOF variants carried by individuals in agricultural versus traditional foraging populations. We did detect a significant difference in the Philippine populations, however the direction was the reverse direction of our predictions, i.e. higher LOF levels in traditional foragers. Overall, we found that geographic region and not subsistence explained most of the variance in LOF alleles; the total number of LOF variants was higher in the Ugandan populations, while the highest LOF homozygosity was found in Filipino individuals. While it is possible that our study is underpowered, this pattern is consistent with other studies of LOF variants across the genome and is likely associated with serial founder effects following an Out of Africa migration^43,44^. Thus, drift alone may explain much of the overall distribution of *OR* and *TASR* LOF variants. While previous studies found higher numbers of functional *OR*s in forager populations^29,36^, these studies were primarily conducted on geographically disparate populations. Our approach permitted a more direct test by comparing populations that are geographical neighbors but diverged in their traditional subsistence strategies.

Our findings have potential implications for understanding the plasticity and development of odor identification. A number of language studies have found that rainforest foragers have a much richer vocabulary of olfactory terms and are better at naming odors than neighboring agriculturalists^30–32^. If our results are generalizable to other foraging populations, it would suggest that these differences in odor vocabulary and identification are not due to physiological differences in the number of different types of olfactory receptors present in foragers. Instead, our results support the Whorfian hypothesis that culture and language can dramatically shape our sensory perception^45^. A similar effect of language has been observed on color perception and has been the subject of vigorous debate in language and cognition^46^. Consistent with our results, a 2022 study investigated perceptions of odor pleasantness across nine cultures (including rainforest foragers and agriculturalists) found that ~95% of the variation in pleasantness rankings was associated with individual taste and odorant molecular identity, suggesting a “universal bedrock of olfactory perception” across populations^47^.

We also identified substantial inter-individual variation in LOF variants, consistent with previous reports of individualized chemosensory gene repertoires and a highly personalized sense of smell^48–52^. While understanding of *TASR* variation is growing, in part because of successful laboratory assay systems (e.g. ref. ^53^), the functional consequences and specific phenotypes associated with *OR* variation are hard to anticipate because ligands of *OR* genes are poorly resolved^49^. *OR* genes represent one of the largest gene families occurring among mammals and are well known for their rapid pace of evolution^24^. Future developments in the in vitro expression and assay of *OR* genes and developments in behavioral assays of characterized individuals will hopefully contribute to phenotypic characterization of this fascinating chemosensory gene family.

Differences in food acquisition methods have long been hypothesized to shape human sensory variation^7,8,14,54^. We identified genetic variation at 26 genes as potential candidates for local adaptation within each geographic region. We did not find evidence of convergent evolution on specific SNPs across continents. However, this is perhaps unsurprising, given the vastly different plant assemblages, cultivars, and regional flavors characterizing these geographic regions. Interestingly, 16 of the 26 genes (~61%) have been directly linked to food perception or feeding behavior in humans or other animals (e.g., food intake, obesity predisposition, BMI), and 5 other genes are associated with traits often linked with BMI and food intake (“age at menarche”, “comparative body size at age 10”, “height”) (Supplementary Table S6). Taken together, ~81% of the genes identified as being candidates for differential selection between agriculturalists and traditional foragers are associated with food intake in some way. These results may reflect convergent responses to different adaptive pressures associated with food availability between rainforest farming and hunting and gathering. Rainforests are considered food-limited environments for humans, and the short-statured small body size phenotype may be a convergent adaptation to these conditions^55–57^. Because olfaction plays an important role in appetite regulation^58^, appetite-regulating *OR*s may be particularly important for rainforest hunter-gatherers in food-limited environments.

Many of the genes we identified (15 of 26) were detected in previous positive selection scans across human populations^28,59–61^ and are promising candidates for comparative analyses. We chose one candidate *OR* haplotype (*OR2A5*) and one *TASR* haplotype (*TAS2R3/4/5*) to explore here in greater detail. Both haplotypes exhibited signatures of positive selection in the BaKiga agriculturalists relative to the Twa and Sua and are directly associated with food preferences relevant in an agricultural context (i.e., fruit intake, and bitter perception of ethanol and alcohol consumption, respectively). Specifically, we found an increased frequency of the haplotype of *OR2A5* associated with fruit intake and the *TAS2R3/4/5* haplotype for reduced bitter perception of ethanol in the BaKiga. The increased frequencies of these haplotypes in the BaKiga may reflect different food histories, including the important cultural role of fermentation (such as fermented drinks like obushera) to Ugandan agricultural societies^62^. These two haplotypes may be particularly promising candidates to explore in adaptations to agriculture in other regions. Both also tended to be higher in the Manobo compared to Philippine foraging communities, although the differences were not as clear. Additionally, both loci have been identified in positive selection scans in dogs^63^, which - along with other domesticated animals - can exhibit convergent adaptations to agricultural human societies living in similar environments^64–66^. However, while selection pressures on *OR* and *TASR* genes are often attributed to foods, it is important to recognize other factors potentially influencing selection on these genes. For example, humans are sensitive to socially and biologically relevant odors^67^. Further, olfactory and taste receptors also act as “extrasensory” nutrient/toxin detectors in other body systems, such as the gastrointestinal, respiratory, and immune systems^68^ (extrasensory chemosensors are discussed further in Supplementary Note 4).

Interestingly, our data suggest that variation at both the *OR2A5* and *TAS2R3/4/5* loci is relatively ancient. For example, the age of the derived *TAS2R3/4/5* haplotype (for decreased ethanol bitterness) is estimated at 619 kyr. The ages of the derived haplotype for five of the six haplotypes we explored in more detail pre-date the origin of modern humans^69^. These data suggest that many of the variants that exhibit allele frequency variation between agriculturalist and traditional foraging populations evolved from standing variation, and their initial emergence and maintenance were not associated with agriculture. Collectively, it is plausible that genetic variation that shapes human perception has mostly evolved prior to out-of-Africa migrations and their allele frequencies were responsive to environmental and cultural adaptations, similar to the model described for genetic variation shaping skin pigmentation^70,71^.

## Methods

### Collaborations and ethics

The study is part of a larger project that seeks to understand human adaptations to rainforest habitats, which is conducted in collaboration with Makerere University and University of the Philippines, Diliman under formal agreements or memoranda of understanding, and approved by the Research and Ethics Committee, Makerere University Faculty of Medicine (protocol 2009-137), the Uganda National Council for Science and Technology (permit HS 617), the National Commission on Indigenous Peoples (NCIP), and the Department of Environment and Natural Resources (permit 03-2010). Consent to saliva collection (Oragene Saliva Collection Kit; DNA Genotek, Ottawa, Canada) and genetic analysis occurred at three levels––local government units, indigenous elder members of each settlement or community, and adult individuals––and in close coordination with the Batwa Development Programme, a nongovernmental cultural organization, or regional officers of the NCIP (Region 2, Barbara Garcia; Region 13, Villarica Lumancas). Our protocol for informed consent was also approved by the Committee for the Protection of Human Subjects, Dartmouth College (protocol 22410), the Institutional Review Board, University of California, Santa Cruz (protocol HS0801367), and the Institutional Review Board, University of Chicago (protocol 16986A).

### Sample donation

Saliva samples were donated by 158 individuals (Fig. 1) and we extracted genomic DNA following protocols of DNA Genotek, Inc. (Ottawa, Canada) in the Anthropological Genomics Laboratory at Pennsylvania State University. DNA samples were standardized to contain at least 10–25 mg of DNA in each tube. Genomic libraries were constructed by the University of Texas at Austin’s Genomic Sequencing and Analysis Facility.

### Target capture and sequencing

We designed custom RNA baits (MYbaits; Arbor Biosciences, Ann Arbor, MI) using autosomal DNA sequence coordinates (Supplementary Data S2) of 840 functional and pseudogenized *OR* genes collected from the Human Olfactory Data Explorer (HORDE)^72^ (https://genome.weizmann.ac.il/horde/), *TASR* gene coordinates from Ensembl (3 *TAS1R*s, 30 functional and pseudogenized *TAS2R*s), and 69 intergenic regions in the human genome from autosomes using the Neutral Region Explorer^73^. We performed target capture on genomic libraries using the MYbaits v3 protocol. In brief, we prepared our extracted DNA samples by introducing a hybridization buffer in which RNA baits were added, then introduced a “blocker” mix to allow blockers to hybridize with DNA segments of interest. We denatured our genomic DNA libraries (95 °C, 5 min) and added RNA baits to the libraries for hybridization (16–24 h). After RNA baits were hybridized with DNA libraries, we recovered target DNA by introducing streptadavin-coated magnetic beads to attract the targeted RNA bound to the baits. We initially washed bead-bound libraries with a solution of hybridization reagent, nuclease-free water, and a wash buffer. The libraries were then placed with washing buffer in a water bath (95 °C, 45 min). A magnetic particle collector was used to pull the beads to the bottom of the tubes, after which supernatant was removed. A binding buffer was introduced to the beads, beads were incubated (95 °C, 10 min) resulting in two more steps of “washing” the streptadavin-coated magnetic beads to remove non-targeted library material from the sample. The enriched libraries captured by the magnetic beads were suspended in 30 uL of 10 mM Tris-CI 0.05% TWEEN −20® solution. The captured DNA was amplified at 14 cycles (Activation: 98 °C, 2 min; Denaturation: 98 °C, 20 s; Annealing: 60 °C, 30 s; Extension: 72 °C, 45 s; Final Extension: 72 °C, 5 min; End: 8 °C, ∞) for sequencing. The resulting captured DNA was sent to the University of Calgary’s Core DNA Services for four lanes of sequencing on Illumina’s NextSeq platform.

### Variant filtering

For each individual, we marked and removed PCR duplicates with *Picard Tools* (http://broadinstitute.github.io/picard/) and aligned reads to the hg19 reference genome using GATK’s *HaplotypeCaller*^74^ to generate individual g.vcf files. We next generated a multi-sample g.vcf file by first using GATK’s *CombineGVCF* to generate a combined g.vcf for all individuals within each population, then *GenotypeGVCF* (settings: stand_call_conf = 30, -stand_emit_conf = 20) to generate a single g.vcf file from the six populations. Using *vcftools*^75^, we filtered all variants by site quality (>30), genotype quality (>20), individual coverage (>50% of sites called/individual), and site coverage (>75% of individuals called/site). We excluded variants on sex chromosomes and variants from four *OR* genes that have been found to have issues mapping to the reference genome. Three of these genes (*OR4C3, OR4C4P, OR4C5*) are duplicated at a separate location of the chromosome in non-reference genomes but not in the hg18 reference^76^, while *OR8U1* is suggested to have been recently formed by a deletion event that fused parts of *OR8U8* and *OR8U9* in some individuals^49,77,78^.

### Validation and quality control

To assess our capture method and potential biases in capture efficacy across different genomic regions that we targeted, we conducted a series of validation and quality checks. These steps are important because both *OR*s and *TASR*s reside in regions rich in segmental duplications. Moreover, these gene families are known to be copy number variable among human populations^77,79^. Even when there is no copy number variation, segmental duplication regions present difficulties because the probes can bind to multiple copies. These regions can also be problematic when sequence reads are aligned, and unique mapping may not be possible^80^. Because of these factors, we expect the read-depth of these areas to be lower than expected across all individuals. Further, it is plausible that our capture platform may be biased in the capture efficacy, potentially introducing technical noise. We would expect unusually high or low read-depth in all samples in such loci. To address these issues, we calculated read-depth using samtools-1.9^81^ (http://www.htslib.org/doc/samtools-depth.html). First, we screened our data for individuals that have consistently higher read-depth for all loci (Supplementary Figs. S2 and S3). These likely indicate a bias in the capture step for these particular samples. Thus, we excluded 25 such individuals (out of 158 samples) from subsequent analysis. The average read-depth of the remaining samples was 97.4. We next filtered genes in our dataset with unusually low read-depth (<20th percentile read-depth) among all the captured genes (Supplementary Figs. S2 and S4). These loci indicate regions where our capture consistently failed. Following all read-depth filtering (Supplementary Fig. S2), our final analysis dataset includes 51 neutral regions, 808 ORs, and 32 TASRs for 133 individuals (21 Agta, 26 BaKiga, 18 Mamanwa, 24 Manobo, 21 Sua, and 23 Twa individuals).

### Variant functional annotation

For each SNP and indel in targeted chemosensory genes and intergenic regions, we used Ensembl’s Variant Effect Predictor (VEP) web server for GRCh37^82^ to identify the nearest gene (or “intergenic”), gene type (protein-coding, segregating pseudogene, or pseudogene), consequence (e.g., missense, synonymous, stop-gain, stop-lost, splice, intronic, 5’ UTR, 3’ UTR, regulatory, downstream, or upstream), protein position, amino acid, and known variation (RefSNP rsID). For variants where VEP provided more than one annotation due to the presence of multiple transcript isoforms, we followed four steps to select a single annotation: (1) coding region annotations (exonic/intronic/UTR) were prioritized over upstream or downstream; (2) exonic annotations were prioritized over intronic or UTR; (3) sensory genes were prioritized over RNA genes; (4) if multiple transcripts per gene were present, the longest transcript was chosen. *OR* segregating pseudogenes are fairly common in human populations^48–50,83,84^. These genes are annotated as a “pseudogene” by VEP. Using data from Olender et al.^48^ and the Human Olfactory Data Explorer (HORDE^72^), we documented the variants that “rescue” these genes to an active status again and included these variants in our analysis of the number of loss-of-function variants (see below). We also found one *OR* gene (*OR52B1P*) that was classified as “protein-coding” by VEP. However, multiple other sources, including Hughes et al.^84^, HORDE, the University of California Santa Cruz Genome Browser^85^, and Ensembl GRCh38 consider it a pseudogene, so we categorized it as a pseudogene.

### Chemosensory and “putatively neutral” variant datasets

To evaluate signatures of positive selection on chemosensory genes, we first filtered our SNPs into “chemosensory” and “putatively neutral” datasets. For the *chemosensory dataset*, we filtered SNPs to only include variants in exons and untranslated regions of functional chemosensory genes (coding and segregating pseudogenes). For the *putatively neutral dataset*, we included biallelic SNPs from targeted intergenic regions and pruned for sites in linkage disequilibrium (*r*^2^ threshold = 0.5) using *plink*^86^ following Schweizer and colleagues^87^. We also included in this putatively neutral dataset SNPs from chemosensory regions that were classified by VEP as intergenic, upstream/downstream of functional genes/pseudogenes, introns of pseudogenes, or ancient chemosensory pseudogenes that are presumed to have experienced a long history of neutral evolution. These ancient pseudogenes include 90 *OR* pseudogenes with premature stop codons shared between modern humans and chimpanzees^84^, and 2 *TASR* pseudogenes (*TAS2R63P, TAS2R67P*) that have been psuedogenized since at least the last common ancestor of hominids (chimpanzees, bonobos, humans)^21,88,89^. After these filtering steps, we were left with a *chemosensory dataset* of 4913 SNPs in the exons and untranslated regions of 378 *OR*s, 2 sweet/umami taste receptor genes (*TAS1R1, TAS1R2*), and 24 bitter taste receptor genes (*TAS2R*s). The *putatively neutral dataset* included 1704 SNPs.

### Assessing overall genetic variation shaped by demographic history

In order to understand overall genetic variation shaped by demographic history, we calculated genome-wide nucleotide variation (**π**) using the –site-pi option in *vcftools*^75^ for the 232 SNPs from targeted neutral intergenic regions with adequate read-depth in each of the six populations: the BaKiga, Sua, and Twa from Uganda, and the Agta, Mamanwa, and Manobo from the Philippines. We then took the **π** values for each site and reported the average genome-wide value for each population (Supplementary Fig. S1).

### Assessing relaxation of selective constraint: potential loss-of-function (LOF) variants

In order to test for a relaxation in selective pressure on chemosensation with agriculture, we specifically targeted potential LOF variants (SNPs, indels) in our chemosensory dataset. We considered SNPs/indels as potential LOF variants by VEP consequence (“stop gained”, “start lost,” “stop lost”, or “frameshift variant”), or if they were previously identified in published lists of pseudogenizing *OR* in HORDE or published sources^83^. We also used ALoFT^90^ to identify frameshift indels that introduce downstream premature stops. We also documented fifteen “rescue” variants (all affecting *OR* genes). Overall, we documented 235 LOF variants among 161 *OR* genes, and 17 LOF variants among 12 *TASR* loci (Supplementary Data S1). We then calculated the number of heterozygous and homozygous LOF mutations in each individual in our sample set. Under relaxed selection, we would expect a higher number of homozygous LOF variants, on average, in agriculturalists relative to traditional foraging populations in both the Philippines and Uganda. While the presence of LOF variants and their heterozygosity are directly affected by effective population sizes, our geographic-neighbors approach provides a less biased comparison. We first performed one-tailed Wilcoxon rank-sum tests to compare the number of homozygous LOF variants carried by individuals in traditional foraging versus agriculturalist populations in each continent. Next, we performed a generalized linear mixed effects model with a Poisson distribution, the individual count of homozygous LOF genes as the dependent variable, subsistence history as a fixed effect, the individual’s count of called LOF genes as an offset variable (controlling for individual variation in coverage), and population of origin nested in continent as random effects. The model was performed in R using the lme4 package^91^ (version 1.1-26).

### Assessing relaxation of selective constraint: Tajima’s D

To further test for different levels of negative selection acting on agricultural and rainforest foraging populations, we calculated Tajima’s D^40^ across 1 kb windows including chemosensory genes for which we have more than 3 SNPs genotyped in all individuals. We also calculated Tajima’s D across whole chromosomes for putatively neutral SNPs. This test measures deviations from the expected allele frequency distribution with negative values indicating selection. Thus, if the strength of purifying selection on chemosensory genes in traditional foraging populations has been stronger than in agriculturalist populations, we expect Tajima’s D values to be smaller in the former than the latter populations when compared to neutral expectations. We used one-tailed Wilcoxon tests to compare Tajima’s D values between chemosensory SNPs and putatively neutral intervals within each population.

### Assessing positive selection and local adaptation: population branch statistics

We used population branch statistics (PBS)^41^ to investigate potential signatures of positive selection on chemosensory genes that aligned with the transition to agriculture or with traditional hunting and gathering in each region. This test assumes that divergent selective pressures acting on different populations will lead to changes in allele frequencies and higher differentiation between populations. PBS measures the relative phylogenetic distances between three populations. Two are the focal populations that we compare to each other and the third is a more distantly related population. Under neutrality, the ratio of phylogenetic distance between these two populations when normalized by the outgroup population should remain constant independent of mutation rate in this locus. Thus, any significant deviation from this expectation may indicate a non-neutral increase in the allele frequency and hence a local adaptation^41,92^. We calculated PBS for exonic and putatively neutral SNPs for each pairing of agricultural (AGR) and hunter-gatherer (HG) populations in each geographic region, treating one HG population from the other continent as the outgroup population (Agta for Ugandan analyses, Twa for Philippine analyses). Using another HG population as the outgroup rather than an AGR population can better identify candidate genes specifically distinguishing agriculturalists from rainforest HGs. For each population pair, we estimated F_st_^93^ using *vcftools* between the AGR population and the HG population, the HG and the outgroup, and the AGR and the outgroup. We omitted SNPs that were invariant within each pair of populations. Any “undefined” F_ST_ values between one population and the outgroup (due to shared fixation of one of the alleles) was set at 0. We then estimated PBS for the agricultural branch. We set negative PBS values as 0. We performed separate analyses for each focal pair in each region, as well as a third analysis that grouped the two HG populations in the region as a single “population.” We identified outlier SNPs for each focal pair using an empirical p-value approach. Specifically, we calculated a percentile for each exonic SNP relative to the presumed neutral SNPs for that focal pair using the *ecdf* function in R, then corrected for multiple testing using the false discovery rate. We identified outliers as those SNPs with an adjusted *p*-value < 0.05. To investigate positive selection associated with rainforest foraging, we repeated these analyses, changing the outgroup to the AGR population from the other continent (e.g., Manobo for Ugandan analyses, BaKiga for Philippine analyses). In total, we performed six analyses per region (three of them using HG as derived, three using AGR as derived). The final number of SNPs for each focal pair was (exonic, presumed neutral): Manobo vs. Agta (2110, 729); Manobo vs. Mamanwa (2008, 680); Manobo vs combined Agta/Mamanwa (2208, 755); BaKiga vs. Sua (3999, 1365); BaKiga vs. Twa (4012, 1372); and BaKiga vs. combined Sua/Twa (4373, 1513).

### Phenotype association of SNPs identified through PBS and linkage disequilibrium analyses

To understand the potential functional effects of the putatively adaptive SNPs, we conducted a focused analysis of the potential functional effects of the 43 SNPs identified as outliers in the PBS analyses by conducting a PheWAS search in GWAS Atlas^94^ and UK Biobank datasets (http://geneatlas.roslin.ed.ac.uk/). In addition, we performed literature searches on each SNP to identify previous work that may link any of these SNPs to functional or evolutionary patterns. We noticed that these SNPs are not randomly distributed across the genome but cluster together and have similar allele frequencies, suggesting that they are in linkage disequilibrium. Specifically, we focused on six haplotype blocks that show significant (*p* < 10^−5^) association with perception-related phenotypes. We further managed to cluster the SNPs from the same genomic locations and with similar association signals into haplotype blocks by showing linkage disequilibrium to each other using *vcftools* with *r*^2^ values > 0.65.

### Haploblock evolutionary history analysis

To investigate the evolutionary history of the select haplotype blocks, we further analyzed them within the broader context of global human population-level data obtained from 1000 Genomes Phase 3 dataset. We visualized the allele frequency distributions for the haplotypes using the R package *rworldmap*^95^. PheWAS associations plots were taken directly from GWAS Atlas. Phylogenetic networks were constructed from phased SNP calls from the 1000 Genomes Project^96^ as well as Neanderthal^97^ and Denisovan^98^ variant calls. We also included Chimpanzee haplotype from the reference genome (PanTro4). In order to integrate data from our own study and 1000 Genomes, we used Illumina HumanOmni1-Quad genotyping array data for the same Twa and BaKiga individuals that we sequenced in this study available from an earlier work^56^. In brief, the data from this array based study allowed us to extend our linkage disequilibrium analysis to non-coding and intergenic sequences, allowing us to better determine the extent of the haplotype blocks and directly compare with the haplotypic variation in 1000 Genomes dataset. We used *plink*^86^ and *vcftools* to extract our haplotype SNPs from this dataset. We used *Picard* to perform a liftover from hg18 to hg19 on the BaKiga/Twa vcf files. We phased our.vcf files without a reference dataset using *Beagle 5.1*^99^. From there we combined the SNP data sets from 1000 Genomes and Twa/BaKiga allowing us to delineate distinct haplotype groups (Supplementary Fig. S5). The alignments were compiled using *VCFtoTree*^100^ and visualized using *PopART* (Population Analysis with Reticulate Trees)^101^. Haploblock lengths used in tree construction included 21,974 bp (Fig. 5C) and 50,142 bp (Fig. 5F). We used the Human Genome Dating database^102^ to estimate the age of the SNP in highest linkage disequilibrium for each of the select haploblocks.

### Statistics and reproducibility

Most statistics in this study (***π***, Tajima’s D, *F*_*st*_, linkage disequilibrium) were calculated in *vcftools* from g.vcf files, while statistical analyses (Wilcoxon rank sums tests, generalized linear mixed effects models and population branch statistics) were performed in R (version 4.0 and above). The specific details of the methods and sample sizes for target capture, variant filtering, quality control of individuals and variants, variant annotation, nucleotide diversity, statistical analyses for assessing relaxation of selective constraint and local adaptation, and PheWAS and haploblock analyses are described in the methods above.

### Reporting summary

Further information on research design is available in the Nature Portfolio Reporting Summary linked to this article.

## Supplementary information

Supplementary Information

Reporting Summary

## Data Availability

All capture/sequencing and g.vcf data has been deposited in the European Genome-Phenome Archive (study id: EGAS00001007307). All other data, including LOF variants (Supplementary Data S1), target coordinates for MYbaits (Supplementary Data S2), PBS values for each pair of focal populations, and data for main figures are available at https://github.com/GokcumenLab/data. An expanded discussion of LOF variant distribution and evolutionary history for two haplotypes of particular interest, methodological figures (e.g., quality control filtering), and lists of significant PBS SNPs per geographic region, PheWAS results, and summaries of other functional annotations and their references are provided in the Supplementary Information.

## Code availability

We provide the R code and input files that we used for visualizations and statistical analysis at https://github.com/GokcumenLab/data.
