# Supplementary material for: Human subsistence and signatures of selection on chemosensory genes
Source: Commun Biol. 2023 Jul 3;6:683. doi: 10.1038/s42003-023-05047-y (PMC10317983; doi:10.1038/s42003-023-05047-y)
Supplement: Supplementary file 1 — Supplementary Information [file 42003_2023_5047_MOESM1_ESM.pdf]

Supplementary Material

Human subsistence and signatures of selection on chemosensory genes

Carrie C. Veilleux, Eva C. Garrett, Petar Pajic, Marie Saitou, Joseph Ochieng, Lilia D. Dagsaan, Nathaniel J. Dominy, George H. Perry, Omer Gokcumen, Amanda D. Melin

Contents

Table of Contents

Supplementary Notes 2

Note 1. Population histories and subsistence economies....2

Note 2. Distributions of segregating LOF variants....3

Note 3. Two haplotypes of particular interest with regards to sensory phenotypes and food intake 3

Note 4. Potential non-dietary influences on patterns of chemosensory gene variation 5

Supplementary Figures 6

Figure S1. Population and regional measures of nucleotide diversity ($\boldsymbol{\pi}$) 6

Figure S2. Schematic illustrating quality check filtering pipeline based on read-depth 7

Figure S3. Normalized read-depth (RD) from captured short-read sequences across different samples for a set of *OR* genes....8

Figure S4. Read-depth (RD) heat map of genes that show low normalized read depth 9

Figure S5. An example of the integrated haplotype-level analysis of region of interest....10

Figure S6. The expression quantitative loci analysis of rs2961144 and its effect on *ARHGEF35* expression 11

Supplementary Tables....12

Table S1. Genes with outlier SNPs in Ugandan PBS analyses and known functional agonists/associations ..12

Table S2. Genes with outlier SNPs in Philippines PBS analyses and known functional agonists/associations 13

Table S3. PheWAS and GWAS results for candidate SNPs in Ugandan populations 14

Table S4. PheWAS and GWAS results for candidate SNPs in Philippine populations 16

Table S5. Summary of haplotype blocks for select outlier PBS SNPs and known functional/behavioral associations....18

Table S6. Diet- and metabolism-affecting candidate genes from PBS analyses 19

Supplementary References 20

Supplementary Notes

Note 1. Population histories and subsistence economies

Three populations of western Uganda participated in this study: the BaKiga, Sua, and Twa. The BaKiga speak Rukiga, a central Bantu language associated with subsistence farming. The BaKiga settled the region ca. 3–2.5 kya[1], and royal court records from Rwanda indicate 500 years of farming at higher elevations[2,3]. The Twa live in close proximity to the BaKiga and speak Rukiga, though their history in the region long predates the Bantu expansion[1,4]. Study participants lived and foraged in Bwindi Impenetrable Forest until the creation of Bwindi Impenetrable National Park in 1991. Today the Twa are dispossessed of their traditional foodways and settled around the perimeter of the park, where they practice subsistence farming[103]. Like the Twa, the Sua express the human pygmy phenotype[5,6], but their ancestry differs. The Sua lived and foraged in Semuliki Forest Reserve, the easternmost extension of the Ituri Forest, until the creation of Semuliki National Park in 1993. The current population of ~100 people lives in isolation on public lands adjacent to the park[7]. Oral testimonies trace their origin to a group of ~10 individuals who emigrated from the Ituri Forest, Democratic Republic of Congo during the 1920s[8]. Cultural affinities with the Efe, also from the Ituri Forest, hint at close relatedness with this well-studied population.

In the Philippines, members of the Agta, Mamanwa, and Manobo participated in this research. The Agta are semi-sedentary foragers who live along the rivers and coastlines of northeastern Luzon. They gather tubers and hunt wild pig, deer, and smaller game in the forests of Northern Sierra Madre Natural Park. Food is also sourced from rivers and ocean littoral zones, as well as trading partners integrated into the cash economy[9–11]. The Mamanwa are sedentary forager-farmers living in the Surigao provinces of northeastern Mindanao. They live today on the edge of arable land, practicing lowland rice farming and/or upland hunting and gathering in the rainforest[12]. The Agta and Mamanwa are sometimes categorized as “negritos”, a colonial-era term that presumes common ancestry on the basis of similar phenotypic traits. Some corroborating evidence exists[13,14], but it is complicated by the deep antiquity (up to 46 kya) of both populations on each island[15]. The Manobo are related to the Sama sea nomads of the Sulu Archipelago, and their arrival on Mindanao (ca. 12 kya) predates the spread of paddy field rice agriculture[15]. Early accounts described the Manobo as swidden farmers[16], but shifts to sedentary farming occurred during the 1940s[17], and it is today focused mainly on rice, corn, and root crops.

Note 2. Distributions of segregating LOF variants

Some segregating LOF variants do show differences between historically agriculturalist and traditional foraging populations within continents (**Fig. 2d**). However, there are only a few such variants and they show inconsistent directionality. For example, a frameshift mutation affecting *OR52E6* has a 33% allele frequency in the communities in Uganda that recently switched from hunting-gathering to subsistence farming lifeways, while no individual from the agricultural BaKiga population carries this allele. Another interesting allele is the rescue variant affecting *OR5R1*. This variant is common in all populations. However, the allele frequency is considerably higher in BaKiga (0.85) as compared to Ugandan traditional foragers (Twa and Sua; 0.55). The trend is reversed in the Philippines, where the same variant is present in higher allele frequencies in traditional foragers (0.84) as compared to the Manobo agriculturalists (0.58).

Note 3. Two haplotypes of particular interest with regards to sensory phenotypes and food intake

a. *OR2A5* and fruit and bread intake

The haplotype encompassing olfactory receptor genes *OR2A5* and *OR2A25* on chromosome 7, spans approximately 22 kb (Hg19: chr7: 143,747,155 - 143,769,129 as calculated from 1000 Genomes dataset), and is of particular relevance to our research focus on diet. From the PheWAS analyses, we identified the ancestral haplotype as related to a preference for "bread intake" ($p<6.7 \times 10^{-7}$) and the derived haplotype associated with increased "fruit intake" ($p<3.5 \times 10^{-12}$) (**Fig. 5a**). This haplotype is represented by 11 linked SNPs present in the whole-exome sequencing data that was conducted on a subset of this population[6]. The agriculturalist population in Uganda (BaKiga) exhibits a relatively high allele frequency of the fruit intake haplotype (0.65) compared to the neighboring Sua (0.15 and Twa (0.45) and the Philippine traditional forager outgroup (Agta: 0.05). Consistently, the Philippine agriculturalists (Manobo) also show increased allele frequency of this haplotype as well (0.10) relative to Agta (0.05) and Mamanwa (0.0), but this difference did not reach significance in PBS analysis for the Philippine region. At the global level, we found that this haplotype also shows a high level of continental allele frequency differences (**Fig. 5b**).

To better understand if the haplotype has recently originated or is the result of standing variation, we investigated the haplotypic variation within this locus (**Fig. 5c**). We found that the fruit intake variant (in blue) is particularly common in Africa and is present in Denisovan but not Neanderthal genomes. It is likely that the fruit intake variant emerged before human-Neanderthal divergence and has remained as

standing variation in the human lineage since then, and may have caused chemosensory perception differences between Neanderthals and Denisovans as well. From a mechanistic perspective, the haplotype block significantly ($p<10^{-10}$) affects the expression of multiple genes, increasing gene expression in some cases, and decreasing it in others. For example, the expression of *ARHGEF35,* which is involved in perception-related GPCR signaling, is substantially decreased (effect size < -0.5) in the presence of the haplotype (**Fig. S6**).

b. *TAS2R3/4/5* and bitterness perception of ethanol and spices

The haplotype encompassing *TAS2R3, TAS2R4,* and *TAS2R5* on chromosome 7 spans 50 Kb (Hg19: chr7: 141,455,780 - 141,505,942) and has been found in published selection scans of African traditional foraging and agricultural populations[18,19]. Previous work linked the six SNPs in this haplotype with perceptual differences and food intake (**Table S1**). The derived haplotype is at a higher frequency in the BaKiga agriculturalist population (~83%) relative to the Sua and Twa traditional foraging populations in Uganda (0.61 and 0.52, respectively; **Fig. 5e**, **Table 1**). It is associated with decreased bitterness perception of ethanol and capsaicin[20] but increased bitterness perception of espresso[21] relative to the ancestral haplotype. This haplotype has also been linked to increased alcohol consumption[22] and adding salt to food (**Fig. 5d**). In contrast, the ancestral haplotype is associated with increased bitter perception of ethanol and capsaicin. In the Philippine populations, the reduced ethanol bitterness haplotype was also at a higher frequency in the Manobo agriculturalists (0.41) relative to the historically foraging Agta (0.28) although the Mamanwa exhibited the highest frequency (0.50). Thus, for three of the four agriculturalist/foraging population pairs, the agriculturalist population was more likely to exhibit the haplotype associated with decreased bitterness perception of ethanol and capsaicin/piperine, increased alcohol consumption, and adding salt to food, while the foraging populations were relatively more likely to exhibit the haplotype associated with increased bitter perception of those compounds. Interestingly, the Mamanwa have a subsistence history as forager-farmers (**Supplementary Note 1**), which may be associated with their high frequency of the reduced ethanol bitterness haplotype.

One of the clear observations from the haplotype networks for this locus (**Figure 5e**) is that the increased ethanol bitterness haplotype (indicated by blue) is highly divergent from the reduced ethanol bitterness haplotypes. Surprisingly, Neanderthal and Denisovan genomes carry the ancestral and derived alleles, respectively, for this locus. The latter is dated to approximately ~619 Kyr (assuming 25 years generation time) and thus predates most of the anatomically modern human migrations. Thus, similar to

the *OR2A5* locus described above, this locus also harbors ancient functional variation that has been segregating among humans for hundreds of thousands of years.

Note 4. Potential non-dietary influences on patterns of chemosensory gene variation

There is growing evidence of the importance of chemosensory receptors as nutrient and toxin detectors in a large number of other tissues and organ systems[23]. *TAS1R*s and *TAS2R*s, for example, are expressed in respiratory epithelia and aid in immunity; allelic variation in *TAS2R38* is associated with differences in sino-nasal infection[24]. *TASR*s in the gastrointestinal tract are involved in glucose sensing and regulating insulin[23,25]. Some *OR*s are expressed in the skin and may play a role in wound healing[26]. In addition, the transition to agriculture is associated with multiple factors that led to increased disease prevalence, including higher population density, close proximity with domesticated animals (and zoonotic disease), and reduced nutritional quality[27,28]. Not surprisingly, genomic studies suggest that the transition to agriculture is associated with positive selection on genes involved in immune function[27,29]. Consequently, it is also important to consider that signatures of an agricultural effect on some *OR*s and *TASR*s may be due to health (e.g., metabolic or immune functions) rather than the odors and tastes of the food itself. Indeed, some studies suggest that the selective pressures influencing variation in *TAS2R38* and *TAS2R16* in African populations may be associated with their extra-sensory roles[30,31]. Overall, a consistent theme is the need for improved genotype-phenotype associates, which will provide important insights into the processes of natural and sexual selection and their interactions with neutral processes in shaping human genetic variation.

Supplementary Figures

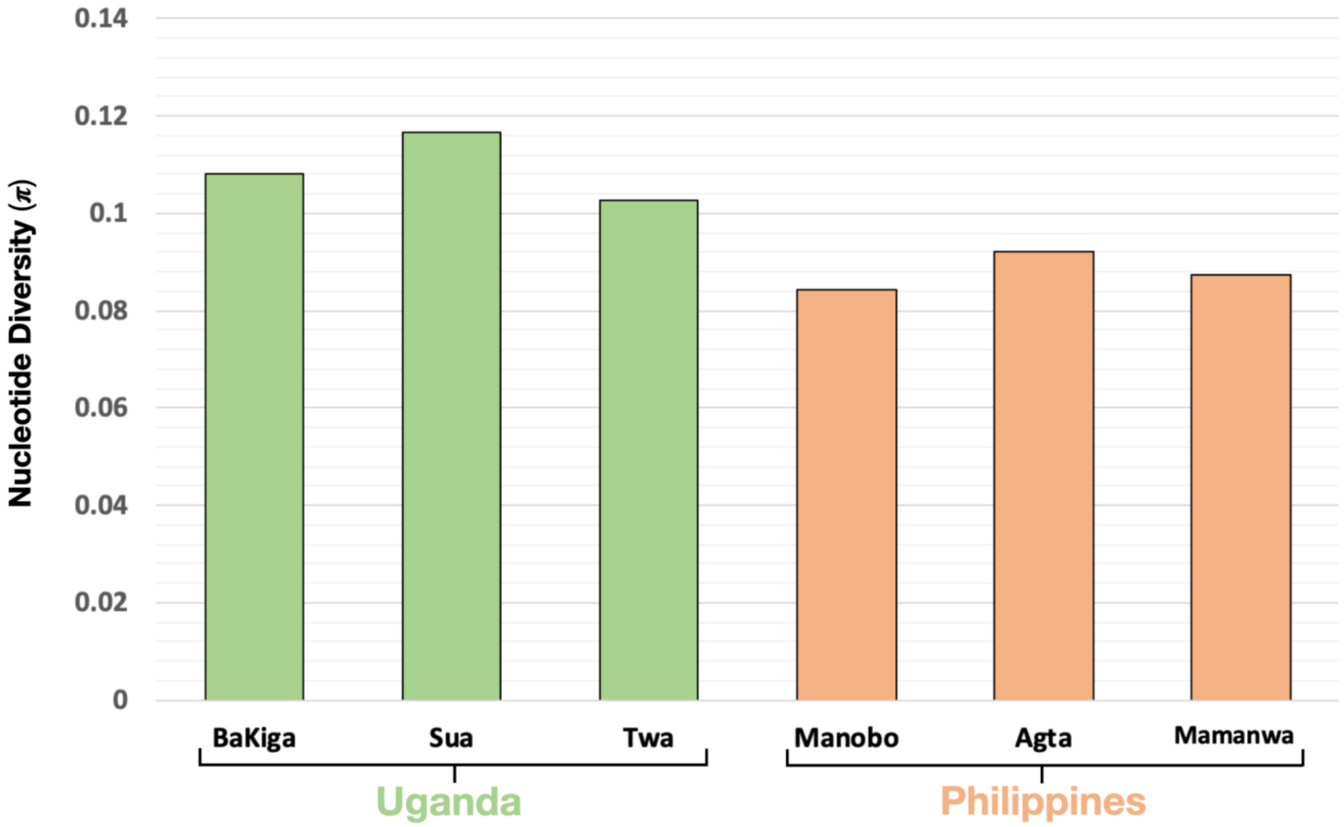

Figure S1. Population and regional measures of nucleotide diversity (π)

Bar plot shows average genome-wide nucleotide diversity (y-axis) calculated for neutrally evolving regions, corresponding to the labeled populations (x-axis) from Uganda (green) and Philippines (orange).

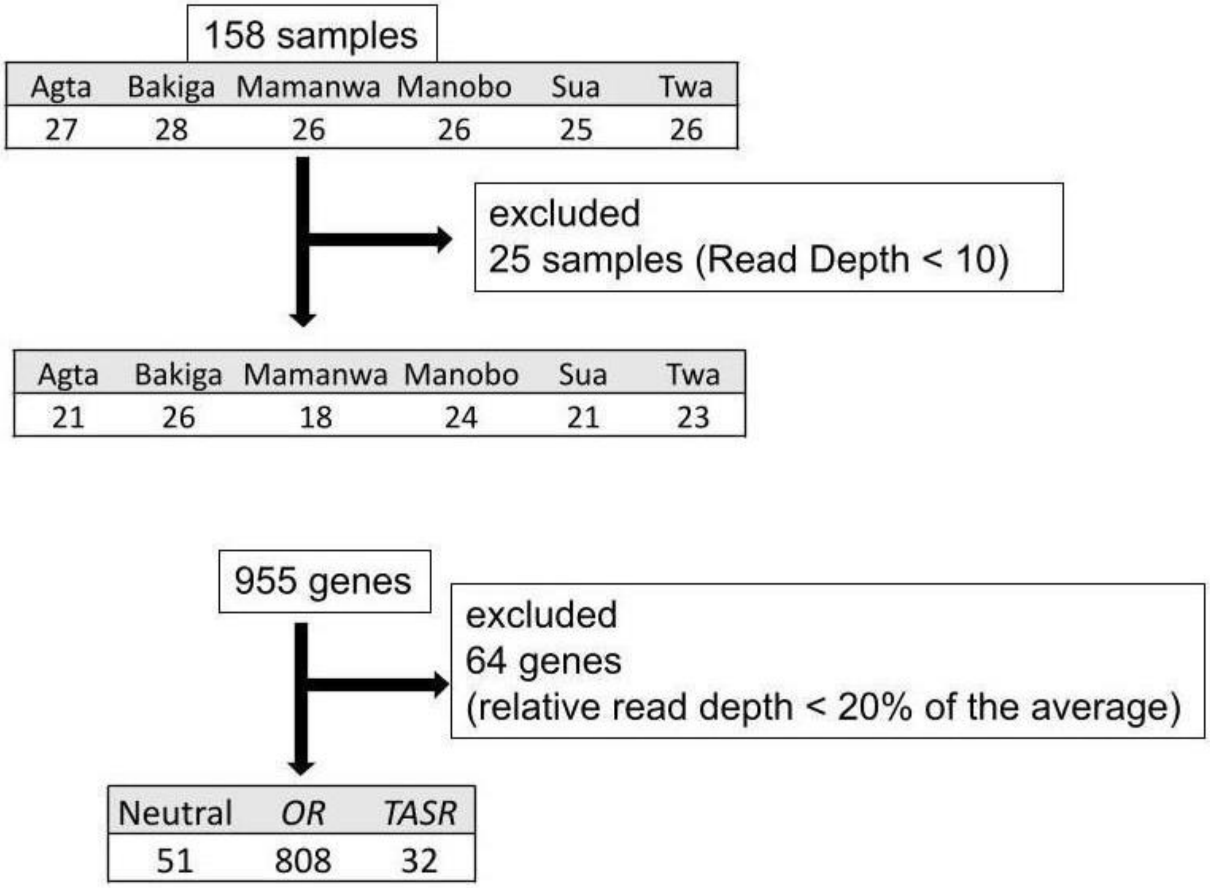

Figure S2. Schematic illustrating quality check filtering pipeline based on read-depth

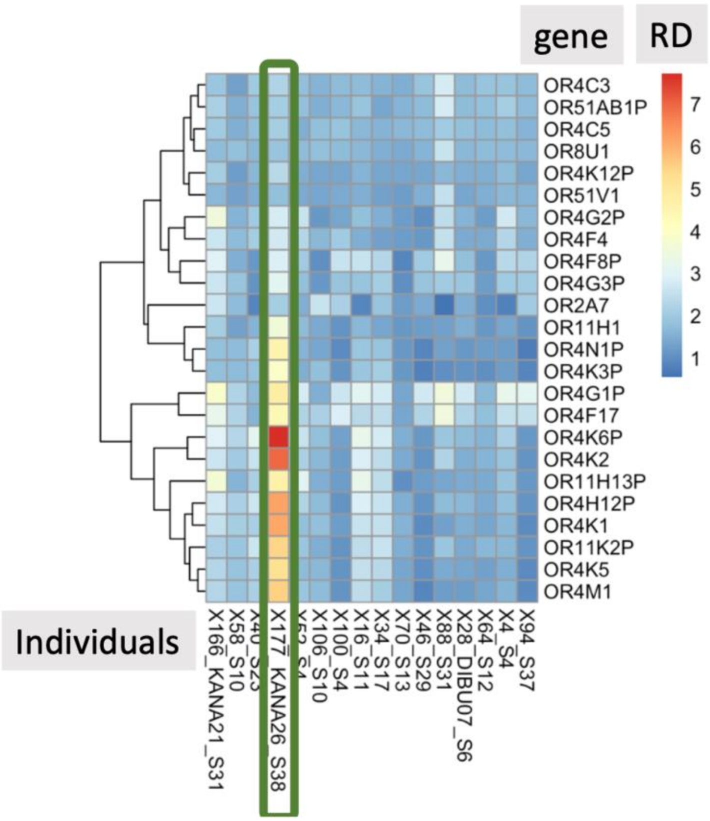

Figure S3. Normalized read-depth (RD) from captured short-read sequences across different samples for a set of *OR* genes

Note that the sample X177_KANA26_S38 (boxed) shows higher read-depth for dozens of olfactory receptor genes as compared to other samples. It is plausible but unlikely that this change in read-depth is due to a large variable duplication. Rather, it is likely that the capture experiment in this sample worked unevenly. Either way, the single nucleotide variant calling for the affected genes in this sample may be enriched for false-positive calls. Therefore, we removed this sample and others with similar read-depth profiles from the downstream analysis.

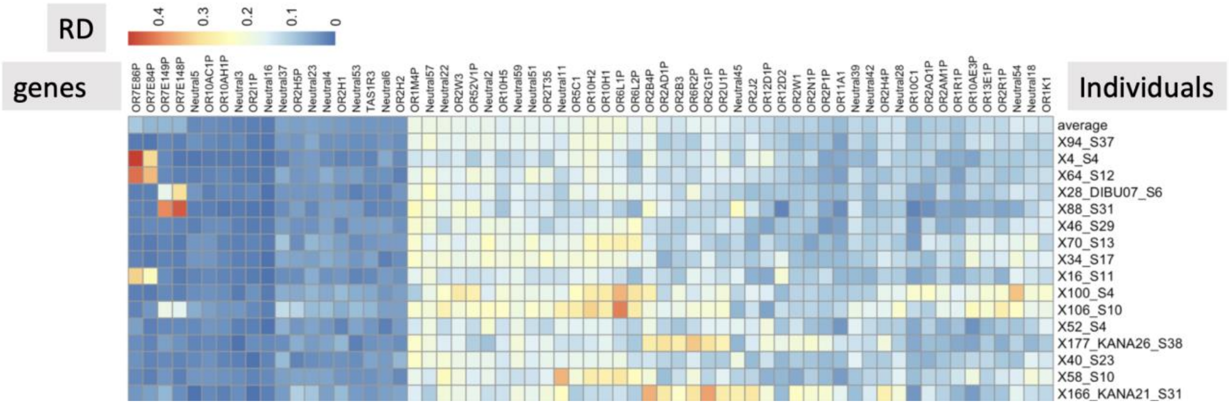

Figure S4. Read-depth (RD) heat map of genes that show low normalized read depth

We note particular genes (on the left side of the heatmap) where the genes show consistently low read-depth across most samples. The most likely reason for this observation is that our platform failed to capture sequences from these regions. Thus, we eliminated these regions from downstream analysis.

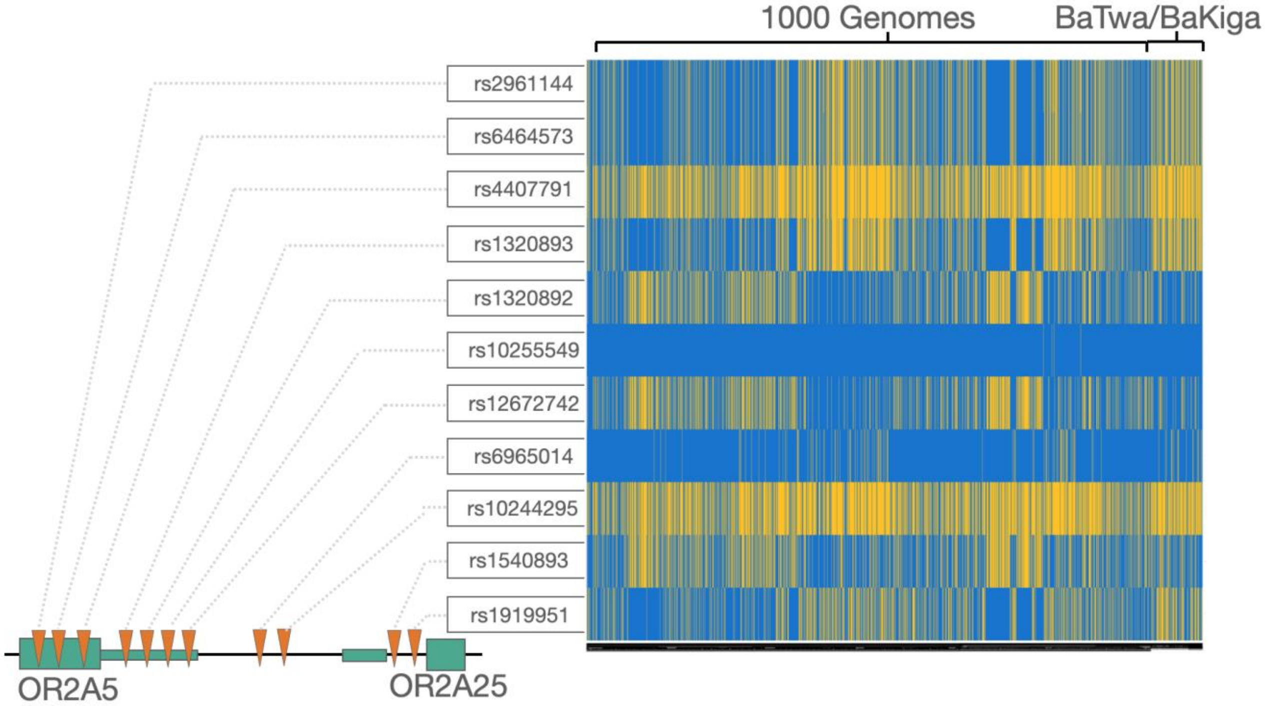

Figure S5. An example of the integrated haplotype-level analysis of region of interest

Briefly, we merged data from our own capture sequencing, data from array-based genotyping data from the same individuals, and 1000 Genomes dataset. The heatmap shows thousands of haplotypes (columns) and allelic variation (in each row).

Tissue	Samples	NES	p-value	m-value
Brain - Anterior cingulate cortex (BA24)	147	-	-	-
Brain - Cerebellar Hemisphere	175	-	-	-
Brain - Cerebellum	209	-	-	-
Brain - Cortex	205	-	-	-
Brain - Frontal Cortex (BA9)	175	-	-	-
Brain - Hippocampus	165	-	-	-
Muscle - Skeletal	706	-	-	-
Whole Blood	670	-0.187	1.6e-4	0.00
Brain - Amygdala	129	-0.355	2.7e-3	1.00
Adrenal Gland	233	-0.373	1.4e-5	1.00
Brain - Caudate (basal ganglia)	194	-0.389	1.5e-5	1.00
Brain - Spinal cord (cervical c-1)	126	-0.391	0.01	1.00
Brain - Substantia nigra	114	-0.398	0.009	1.00
Brain - Putamen (basal ganglia)	170	-0.412	5.9e-5	1.00
Small Intestine - Terminal Ileum	174	-0.431	9.8e-10	1.00
Vagina	141	-0.438	1.4e-8	1.00
Brain - Hypothalamus	170	-0.442	5.5e-5	1.00
Heart - Left Ventricle	386	-0.450	1.0e-8	1.00
Breast - Mammary Tissue	396	-0.451	1.8e-20	1.00
Uterus	129	-0.457	9.2e-4	1.00
Artery - Tibial	584	-0.463	1.5e-20	1.00
Stomach	324	-0.480	8.2e-18	1.00
Colon - Transverse	368	-0.488	8.1e-23	1.00
Cells - EBV-transformed lymphocytes	147	-0.509	1.2e-5	1.00
Brain - Nucleus accumbens (basal ganglia)	202	-0.514	3.2e-8	1.00
Prostate	221	-0.519	6.7e-11	1.00
Esophagus - Gastroesophageal Junction	330	-0.524	1.6e-11	1.00
Colon - Sigmoid	318	-0.528	2.2e-14	1.00
Nerve - Tibial	532	-0.558	4.5e-20	1.00
Esophagus - Muscularis	465	-0.559	6.5e-18	1.00
Ovary	167	-0.568	7.3e-6	1.00
Adipose - Visceral (Omentum)	469	-0.589	4.0e-23	1.00
Adipose - Subcutaneous	581	-0.589	2.5e-24	1.00
Heart - Atrial Appendage	372	-0.630	3.4e-14	1.00
Artery - Aorta	387	-0.631	3.8e-19	1.00
Testis	322	-0.634	1.9e-15	1.00
Thyroid	574	-0.634	4.3e-35	1.00
Lung	515	-0.652	1.0e-30	1.00
Artery - Coronary	213	-0.663	1.7e-14	1.00
Liver	208	-0.668	7.4e-12	1.00
Pituitary	237	-0.672	3.6e-13	1.00
Cells - Cultured fibroblasts	483	-0.675	3.1e-30	1.00
Spleen	227	-0.677	2.8e-13	1.00
Skin - Sun Exposed (Lower leg)	605	-0.693	6.7e-43	1.00
Pancreas	305	-0.699	2.8e-18	1.00
Skin - Not Sun Exposed (Suprapubic)	517	-0.700	7.5e-34	1.00
Minor Salivary Gland	144	-0.706	8.6e-9	1.00
Esophagus - Mucosa	497	-0.721	2.0e-34	1.00
Kidney - Cortex	73	-0.732	9.7e-5	1.00

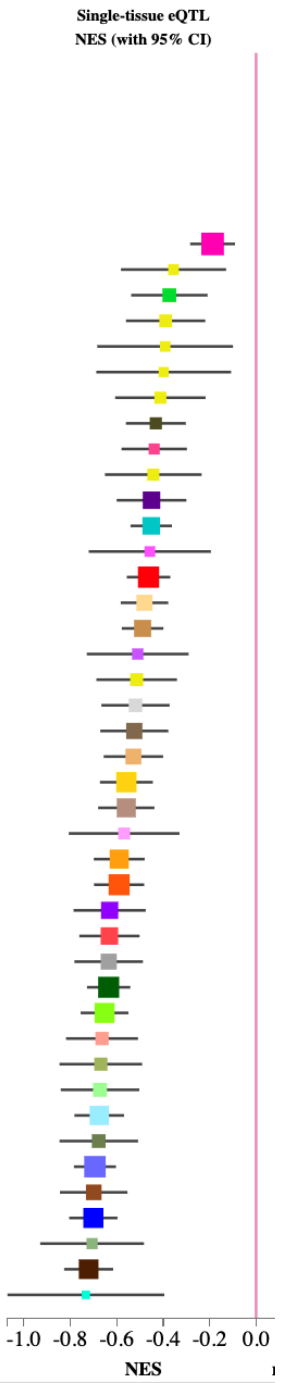

Figure S6. The expression quantitative loci analysis of rs2961144 and its effect on *ARHGEF35* expression

This data and the figure is modified from an GTEx Portal output (gtexportal.org, accessed: March 31, 2021).

Supplementary Tables

Table S1. Genes with outlier SNPs in Ugandan PBS analyses and known functional agonists/associations

PBS results comparing the BaKiga to (1) the Sua, (2) the Twa, and (3) an analysis combining the Sua and Twa into one traditional foraging (TF) population. "X" indicates that SNP(s) in that gene were significant in the analysis of each focal pair of populations. Note that for genes identified in multiple focal pairs, not all SNPs may have been identified in all genes. Known agonists/associations and presence in previous positive selection scans from literature review. Shaded boxes are haploblocks that were targeted for more detailed investigation. Haploblock colours are consistent with Table S3 and Table S7.

			BaKiga &				
**Derived Group**	**Genes**	**SNP(s)**	**Sua**	**Twa**	**TF**	**Known Functional Agonists/Associations**	**Other Scans**
Agriculturalists	*TAS2R3*	rs2270009[S]	x	x	x		
	TAS2R4	rs2233998[m], rs2234001[m], rs2234002[m]	x	x	x	Bitter perception of ethanol, capsaicin, espresso[20,21], alcohol consumption[22]	humans [18,19] dogs[32]
	TAS2R5	rs2234012[5UTR], rs2227264[m]		x	x		
	OR1L8	rs10985703[S], rs10985704[m], rs1999182[S]	x			Unknown agonist	no
	OR2A5	rs2961144[m], rs6464573[m], rs6464574[m]	x		x	Unknown agonist	dogs[32]
	OR2D3	rs11605995[S], rs12806437[m], rs2035844[S]	x	x	x	Food intake in cattle[33,34]	no
	OR14C36	rs28448343[m]	x		x	Obesity predisposition[35]	humans[18]
	OR2Z1	rs28324[S]		x	x	Unknown agonist	no
Traditional Foragers	*TAS2R42*	rs1669413[m], rs1650019[S]	x			Unknown agonist[36]	humans[37]
	OR2L8	rs4925788[m], rs4925583**, rs4925792[m], rs10888281**	x		x	Furfuryl methyl sulfide (onion/garlic), 2-pentylpyridine[38]	humans[19,39]
	OR4D2	rs80043692[m], rs9912728[S]	x			Methylation of gene associated with BMI & food intake[40]	no

For SNPs, superscript indicates functional consequence: synonymous (S), missense (m), 5'UTR (5UTR), and premature stop (**).

Table S2. Genes with outlier SNPs in Philippines PBS analyses and known functional agonists/associations

PBS results comparing the Manobo to (1) the Agta, (2) the Mamanwa, and (3) an analysis combining the Agta and Mamanwa into one traditional foraging (TF) population. "X" indicates that SNP(s) in that gene were significant in the analysis of each focal pair of populations. Known agonists/associations and presence in previous positive selection scans from literature review. Shaded boxes are haploblocks that were targeted for more detailed investigation. Haploblock colors are consistent with Table S4 and Table S6.

Derived Group	Genes	SNP(s)	Agta	Manobo & Mamanwa	TF	Known Functional Agonists/Associations	Other Scans
Agriculturalists	*TAS1R2*	rs68081213	x			sweet compounds	humans[41]
	OR7A10	rs3752195	x			unknown agonist	No
Traditional Foragers	*OR2AE1*	rs2572023^{m}	x			unknown agonist	No
	OR4K15	rs11158071^{S}		x		diallyl sulfide[42]	no
	OR4P4	rs76160133**			x	obesity predisposition in children[43]	humans[19]
	OR5D14	rs297055^{m}			x	Orange[42]	humans[19,39]
	OR5M10	rs10792043^{m}			x	average daily weight gain in cattle[44]	humans[19]
	OR5M11	rs628524^{m}			x	unknown agonist	humans [3]
	OR5R1	rs998544^{m}			x	tropical adaptation in Nellore cattle [29]	humans[19,39]
	OR5T1	rs12360890^{m}			x	unknown agonist	humans[19]
	OR8H1	rs1842674^{S}			x	unknown agonist	humans[39]
	OR8K1	rs10896271^{m}, rs10896272^{m}			x	MCMP (methyl ß-naphthyl ketone, l-carvone, methyl isoeugenol, and phenylethyl acetate)[45]	humans[39]
	OR8J2	rs7130251^{S}			x	unknown agonist	no
	OR7G3	rs10424352^{s}, rs10407484^{s}, rs10414255^{m}		x		rs10414255 -eating behaviors, BMI, and body fat[46]	no
	OR51B4	rs10837771^{S}	x		x	unknown agonist	no

For SNPs, superscript indicates functional consequence: synonymous (s), missense (m), and premature stop (**).

Table S3. PheWAS and GWAS results for candidate SNPs in Ugandan populations

SNPs are organized by whether they are derived in the agriculturalist population or the traditional foraging population. Boxes indicate haplotype blocks across genes identified in this study. Shaded boxes are haploblocks that were targeted for more detailed investigation. Haploblock colors are consistent with Table S1 and Table S7.

Gene	SNP	Position	TraitUK	pUK	GWAS	pGWAS
Derived Group = Agriculturalists						
OR14C36	rs28448343[m]	chr1:248512498	N/A	N/A	Relative age voice broke (male), Cannabis use	0.000048, 8.2e-5
TAS2R3	rs2270009[S]	chr7:141464765	Salt added to food	6.70E-37	Salt added to food	1.10E-27
TAS2R4	rs2233998[m]	chr7:141478308	Salt added to food	1.20E-37	Salt added to food	8.50E-26
TAS2R4	rs2234001[m]	chr7:141478574	Salt added to food	8.60E-40	Salt added to food	5.90E-27
TAS2R4	rs2234002[m]	chr7:141478800	Salt added to food	1.09E-39	Salt added to food	1.03E-26
TAS2R5	rs2234012[5UTR]	chr7:141490107	Salt added to food	9.00E-39	Salt added to food	4.50E-26
TAS2R5	rs2227264[m]	chr7:141490238	Salt added to food	1.90E-37	Salt added to food	7.00E-27
OR2A5	rs2961144[m]	chr7:143747870	Fresh fruit intake, bread intake	2.5e-12, 7.6e-8	Fresh fruit intake, bread intake	3.5e-12, 6.9e-7
OR2A5	rs6464573[m]	chr7:143748098	Fresh fruit intake, bread intake	2.5e-12, 7.6e-8	Fresh fruit intake, bread intake	4.09e-12, 6.65e-7
OR2A5	rs6464574[m]	chr7:143748257	Fresh fruit intake, bread intake, hiatus hernia	2.9e-12, 5.4e-7, 9.6e-5	Fresh fruit intake, bread intake	8.24e-12, 1.1e-6
OR1L8	rs10985703[S]	chr9:125330325	N/A	N/A	N/A	N/A
OR1L8	rs10985704[m]	chr9:125330678	gastro-oesophageal reflux (gord) / gastric reflux	1.50E-03	Coffee Type: instant	6.80E-05
OR1L8	rs1999182	chr9:125330739	N/A	N/A	Coffee Type: instant	6.40E-05
OR2D3	rs11605995[S]	chr11:6942628	N/A	N/A	Neuroticism general factor	4.50E-05

OR2D3	rs12806437[m]	chr11:6942695	N/A	N/A	N/A	N/A
OR2D3	rs203584[Ss]	chr11:6942952	N/A	N/A	Neuroticism general factor	5.30E-05
OR2Z1	rs28324[S]	chr19:8841558	Eosinophil count	1.10E-05	N/A	N/A

Derived Group = Traditional Foragers

OR2L8	rs4925788[m]	chr1:248112745	N/A	N/A	N/A	N/A
OR2L8	rs4925583**	chr1:248112809	N/A	N/A	N/A	N/A
OR2L8	rs4925792[m]	chr1:248112836	N/A	N/A	N/A	N/A
OR2L8	rs10888281**	chr1:248113026	N/A	N/A	N/A	N/A
TAS2R42	rs1669413[m]	chr12:11338781	Coffee, tea intake	1.80E-13	Coffee, tea intake, salt added	2.70E-10
TAS2R42	rs1650019[S]	chr12:11338983	Coffee, tea intake	1.40E-13	Coffee, tea intake, salt added	3.80E-10
OR4D2	rs80043692[m]	chr17:56247306	N/A	N/A	N/A	N/A
OR4D2	rs9912728[S]	chr17:56247454	Myocyte count	9.90E-05	Immunological: CD8:%R5+	3.20E-05

For SNPs, superscript indicates functional consequence: synonymous (s), missense (m), 5'UTR (5UTR), premature stop (**).

Table S4. PheWAS and GWAS results for candidate SNPs in Philippine populations

SNPs are organized by whether they are derived in the agriculturalist population or the traditional foraging population. Boxes indicate haplotype blocks across genes identified in this study. Shaded boxes are haploblocks that were targeted for more detailed investigation. Haploblock colors are consistent with Table S2 and Table S7.

Gene	SNP	Position	TraitUK	pUK	GWAS	pGWAS
Derived Group: Agriculturalists						
TAS1R2	rs68081213[S]	chr1:19184077	Pain and other conditions associated with female genital organs and menstrual cycle	9.90E-05	N/A	N/A
OR7A10	rs3752195[S]	chr19:14951898	N/A	N/A	Ophthalmological: Vertical cup-disc ratio	6.60E-05
Derived Group = Traditional Foragers						
OR2AE1	rs2572023[m]	chr7:99474427	cut off at -08: Platelet distribution width,count,crit/ impedance of amr/ trunk predicted mass	5.50E-15	Lipid::Sterol, Steroid::androsterone sulfate/ Impedance of arm	4.10E-19
OR51B4	rs10837771[m]	chr11:5322737	cut off at -08:RBC dist width/Mean reticulocyte volume/Mean sphered cell volume/ Reticulocyte percentage	3.00E-24	Red cell distribution width	1.10E-10
OR4K15	rs11158071[S]	chr14:20444490	N/A	N/A	N/A	N/A
OR7G3	rs10424352[S]	chr19:9236886	Processed meat intake/Impedance of whole body	1.60E-06	Impedance of whole body	3.10E-05
OR7G3	rs10407484[S]	chr19:9237435	Processed meat intake/Impedance of whole body	1.90E-06	Impedance of whole body	3.30E-05
OR7G3	rs10414255[m]	chr19:9237542	Processed meat intake/Impedance of whole body	1.90E-06	Impedance of whole body/Lipid::Lysolipid::2-stearoylglycerophosphocholine	3.40E-05
OR4P4	rs76160133**	chr11:55406022	N/A	N/A	Cholesterol esters in large VLDL/Total lipids in small VLDL	1.10E-05
OR5D14	rs297055[m]	chr11:55563900	Hypertension/Mean platelet (thrombocyte) volume	2.10E-10	High blood pressure	1.30E-08
OR8J2	rs7130251[S]	chr11:55979266	Comparative body size at age 10	9.80E-06	Male-specific factors - Hair/balding pattern: Pattern 4/ Comparative body size at age 10	1.10E-05

OR5T1	rs12360890^{m}	chr11:56043604	Number of full sisters/ J84 Other interstitial pulmonary diseases	1.00E-05	Age when periods started (menarche) (female)	2.30E-08
OR8H1	rs1842674^{S}	chr11:56058197	Number of full sisters/ J84 Other interstitial pulmonary diseases	1.30E-05	Age when periods started (menarche) (female)	1.80E-08
OR8K1	rs10896271^{m}	chr11:56113575	Number of full sisters/ J84 Other interstitial pulmonary diseases	1.10E-05	Age when periods started (menarche) (female)	2.40E-08
OR8K1	rs10896272^{m}	chr11:56113593	Number of full sisters/ J84 Other interstitial pulmonary diseases	9.60E-06	Age when periods started (menarche)/Metabolic	4.00E-08
OR5R1	rs998544^{m}	chr11:56184888	Number of full sisters/ J84 Other interstitial pulmonary diseases	1.90E-05	Age when periods started (menarche)/Metabolic	4.80E-08
OR5M11	rs628524^{m}	chr11:56310222	hypertension	7.70E-11	High blood pressure	5.60E-07
OR5M10	rs10792043^{m}	chr11:56344993	hypertension/standing height	1.90E-05	height	6.60E-09

For SNPs, superscript indicates functional consequence: synonymous (s), missense (m), 5’UTR (5UTR), premature stop (**).

Table S5. Summary of haplotype blocks for select outlier PBS SNPs and known functional/behavioral associations.

Chr	Position	Linked Genes	Significant SNPs	PheWAS Association	Other Associations	Selection signal:
1	248,112,745 - 248,113,026	*OR2L8*	rs4925788, rs4925583, rs4925792, rs10888281	n/a	Odorants: onion/garlic[a], 2-pentlpyridine	Uganda - foragers
7	143,747,870 - 143,748,257	*OR2A5*	rs2961144, rs6464573, rs6464574	Fresh fruit intake or bread intake	n/a	Uganda - agriculturalists
7	141,464,765 - 141,490,238	*TAS2R3, TAS2R4, TAS2R5*	rs2270009, rs2233998, rs2234001, rs2234002, rs2234012, rs2227264	Salt added to food	Bitterness of ethanol, capsaicin, espresso; alcohol consumption	Uganda - agriculturalists
9	125,330,325 - 125,330,739	*OR1L8*	rs10985703, rs10985704, rs1999182	Instant coffee intake; gastric reflux	n/a	Uganda - agriculturalists
11	6,942,628 - 6,942,952	*OR2D3*	rs11605995, rs12806437, rs2035844	Neuroticism general factor	Food intake in cattle	Uganda - agriculturalists
11	56,043,604 - 56,184,888	*OR5T1*, *OR5R1*, *OR8H1*, *OR8K1*	rs12360890, rs1842674, rs10896271, rs10896272, rs998544	Age at menarche	Tropical adaptation in cattle, Odorant: honey-like floral odor[b]	Philippines - foragers
11	56,310,222 - 56,344,993	*OR5M10, OR5M11*	rs628524, rs10792043	Hypertension	Average daily weight gain in cattle	Philippines - foragers
12	11,338,781 - 11,338,983	*TAS2R42*	rs1669413, rs1650019	Coffee/tea intake	n/a	Uganda - foragers
17	56,247,306 - 56,247,454	*OR4D2*	rs80043692, rs9912728	Immunological, myocyte count	Methylation of gene associated with BMI & food intake	Uganda - foragers
19	9,236,996 - 9,237,542	*OR7G3*	rs10424352, rs10407484, rs10414255	Processed meat intake, impedance of body[c]	Eating behavior, BMI, body fat	Philippines - foragers

Note: haplotype position is based on the locations of the significant SNPs. PheWAS association data from this study (Tables S3, S4). References for other associations are available in Tables S1 and S2.

[a]Onion/garlic odorant: furfuryl methyl sulfide; [b]"Honey-like floral odor" is compound MCMP created by Saito et al.[45] and stimulates *OR8K1*.

[c]"Impedance of body" is a measure associated with body fat.

Table S6. Diet- and metabolism-affecting candidate genes from PBS analyses

List of genes containing SNPs identified in PBS analyses as having highly divergent allele frequencies in either the agriculturalist or traditional foraging populations ("Derived Population"). Functional effects and reference sources are in Tables S1-S4, and have been grouped into three categories: effects on ingestive behaviors, effects on food odor/taste, or effects on body size/development that is associated with diet. Shaded rows match haploblock colors of Tables S1-S4, and dashed boxes indicate genes linked in a haploblock.

	Gene	Derived Population	Ingestive Behaviors	Food Odor/Taste	Body Size/Development Associated with Diet
Philippines	*TAS1R2*	agriculturalist	yes	yes	
	OR7A10	agriculturalist			
	OR2AE1	foraging			
	OR51B4	foraging			
	OR4K15	foraging			
	OR7G3	foraging	yes		yes
	OR4P4	foraging	yes		
	OR5D14	foraging		yes	
	OR8J2	foraging			yes
	OR5T1	foraging			yes
	OR8H1	foraging			yes
	OR8K1	foraging		yes	yes
	OR5R1	foraging			yes[+]
	OR5M11	foraging	yes[+]		
	OR5M10	foraging			yes
Uganda	*OR14C36*	agriculturalist	yes		
Uganda	*TAS2R3*	agriculturalist	yes	yes	
	TAS2R4	agriculturalist	yes	yes	
	TAS2R5	agriculturalist	yes	yes	
	OR2A5	agriculturalist	yes		
	OR1L8	agriculturalist	yes		
	OR2D3	agriculturalist	yes[†]		
	OR2Z1	agriculturalist			
	OR2L8	foraging		yes	
	TAS2R42	foraging	yes		
	OR4D2	foraging	yes**		
			13/26 genes	7/26 genes	7/26 genes

**methylation of *OR4D2* associated with food intake and BMI.

[+]Associations between gene and behaviors observed in cattle.

Supplementary References

1. Patin, E. *et al.* The impact of agricultural emergence on the genetic history of African rainforest hunter-gatherers and agriculturalists. *Nat. Commun.* **5**, ncomms4163 (2014).

2. Ngologoza, P. *Kigezi and its people*. (Fountain Publishers, 1998).

3. Zanika, P. The impact of (forest) nature conservation on indigenous peoples: the Batwa of south-western Uganda: a case study of the Mgahinga and Bwindi Impenetrable Forest Conservation Trust. in *Forest Peoples Programme* 165–194 (2001).

4. Lewis, J. *The Batwa pygmies of the Great Lakes Region*. (Minority Rights Group International, 2000).

5. Perry, G. H. & Dominy, N. J. Evolution of the human pygmy phenotype. *Trends Ecol. Evol.* **24**, 218–225 (2009).

6. Perry, G. H. *et al.* Adaptive, convergent origins of the pygmy phenotype in African rainforest hunter-gatherers. *Proc. Natl. Acad. Sci.* **111**, E3596–E3603 (2014).

7. Frankland, S. Pygmic Tours. *Afr. Study Monogr. Suppl. Issue* **26**, 237–256 (2001).

8. Forno, G. Marginalization and uprooting: the Basua pygmies of the Bundibugyo District. in *Rwenzori: Histories and cultures of an African Mountain* (eds. Pennacini, C. & Wittenberg, H.) (Fountain Publishers, 2008).

9. Griffin, P. B. & Estioko-Griffin, A. *The Agta of northeastern Luzon: recent studies*. (University of San Carlos, 1985).

10. Rai, N. K. *Living in a lean-to: Philippine negrito foragers in transition*. (Museum of Anthropology, University of Michigan, 1990).

11. Minter, T. Ethnology and linguistics : Contemporary relations between Agta and their farming neighbours in the northern Sierra Madre of Philippines. *Senri Ethnol. Stud.* 205–228 (2009) doi:info:doi/10.15021/00002592.

12. Maceda, M. N. *The culture of the Mamanua (Northeast Mindanao) as compared with that of the other negritos of Southeast Asia*. (University of San Carlos, 1975).

13. Reid, L. A. Who are the Philippine Negritos? Evidence from language. *Hum. Biol.* **85**, 329–358 (2013).

14. Jinam, T. A. *et al.* Discerning the origins of the Negritos, First Sundaland People: deep divergence and archaic admixture. *Genome Biol. Evol.* **9**, 2013–2022 (2017).

15. Larena, M. *et al.* Multiple migrations to the Philippines during the last 50,000 years. *Proc. Natl. Acad. Sci.* **118**, (2021).

16. Garvan, J. M. The Manóbos of Mindanáo. *Mem Natl Acad Sci* **23**, 1–265 (1931).

17. Hires, G. A. & Headland, T. N. A sketch of Western Bukidnon Manobo farming practices, past and present. *Philipp. Q. Cult. Soc.* **5**, 65–77 (1977).

18. Lachance, J. *et al.* Evolutionary history and adaptation from high-coverage whole-genome sequences of diverse African hunter-gatherers. *Cell* **150**, 457–469 (2012).

19. Pickrell, J. K. *et al.* Signals of recent positive selection in a worldwide sample of human populations. *Genome Res.* **19**, 826–837 (2009).

20. Nolden, A. A., McGeary, J. E. & Hayes, J. E. Differential bitterness in capsaicin, piperine, and ethanol associates with polymorphisms in multiple bitter taste receptor genes. *Physiol. Behav.* **156**, 117–127 (2016).

21. Hayes, J. E. *et al.* Allelic variation in *TAS2R* bitter receptor genes associates with variation in sensations from and ingestive behaviors toward common bitter beverages in adults. *Chem. Senses* **36**, 311–319 (2011).

22. Choi, J.-H., Lee, J., Yang, S. & Kim, J. Genetic variations in taste perception modify alcohol drinking behavior in Koreans. *Appetite* **113**, 178–186 (2017).

23. Foster, S. R., Roura, E. & Thomas, W. G. Extrasensory perception: Odorant and taste receptors beyond the nose and mouth. *Pharmacol. Ther.* **142**, 41–61 (2014).

24. Workman, A. D., Palmer, J. N., Adappa, N. D. & Cohen, N. A. The role of bitter and sweet taste receptors in upper airway immunity. *Curr. Allergy Asthma Rep.* **15**, 72 (2015).

25. Dotson, C. D. *et al.* Bitter taste receptors influence glucose homeostasis. *PLOS ONE* **3**, e3974 (2008).

26. Busse, D. *et al.* A synthetic sandalwood odorant induces wound-healing processes in human keratinocytes via the olfactory receptor OR2AT4. *J. Invest. Dermatol.* **134**, 2823–2832 (2014).

27. Harper, K. N. & Armelagos, G. J. Genomics, the origins of agriculture, and our changing microbe-scape: Time to revisit some old tales and tell some new ones. *Am. J. Phys. Anthropol.* **152**, 135–152 (2013).

28. Larsen, C. S. The agricultural revolution as environmental catastrophe: Implications for health and lifestyle in the Holocene. *Quat. Int.* **150**, 12–20 (2006).

29. Mathieson, I. *et al.* Genome-wide patterns of selection in 230 ancient Eurasians. *Nature* **528**, 499–503 (2015).

30. Campbell, M. C. *et al.* Origin and differential selection of allelic variation at *TAS2R16* associated with salicin bitter taste sensitivity in Africa. *Mol Biol Evol* **31**, 288–302 (2014).

31. Campbell, M. C. *et al.* Evolution of functionally diverse alleles associated with PTC bitter taste sensitivity in Africa. *Mol. Biol. Evol.* **29**, 1141–1153 (2012).

32. Friedenberg, S. G., Meurs, K. M. & Mackay, T. F. C. Evaluation of artificial selection in Standard Poodles using whole-genome sequencing. *Mamm. Genome* **27**, 599–609 (2016).

33. Olivieri, B. F. *et al.* Genomic regions associated with feed efficiency indicator traits in an experimental Nellore cattle population. *PLOS ONE* **11**, e0164390 (2016).

34. Taye, M. *et al.* Whole genome scan reveals the genetic signature of African Ankole cattle breed and potential for higher quality beef. *BMC Genet.* **18**, 11 (2017).

35. Mariman, E. C. M. *et al.* Olfactory receptor genes cooperate with protocadherin genes in human extreme obesity. *Genes Nutr.* **10**, 16 (2015).

36. Thalmann, S., Behrens, M. & Meyerhof, W. Major haplotypes of the human bitter taste receptor TAS2R41 encode functional receptors for chloramphenicol. *Biochem. Biophys. Res. Commun.* **435**, 267–273 (2013).

37. Sjöstrand, A. E. *et al.* Taste perception and lifestyle: insights from phenotype and genome data among Africans and Asians. *Eur. J. Hum. Genet.* **29**, 325–337 (2021).

38. Ijichi, C. *et al.* Metabolism of odorant molecules in human nasal/oral cavity affects the odorant perception. *Chem. Senses* **44**, 465–481 (2019).

39. López Herráez, D. *et al.* Genetic variation and recent positive selection in worldwide human populations: evidence from nearly 1 million SNPs. *PLOS ONE* **4**, e7888 (2009).

40. Ramos-Lopez, O. *et al.* Associations between olfactory pathway gene methylation marks, obesity features and dietary intakes. *Genes Nutr.* **14**, 11 (2019).

41. Kim, U., Wooding, S., Riaz, N., Jorde, L. B. & Drayna, D. Variation in the human *TAS1R* taste receptor genes. *Chem. Senses* **31**, 599–611 (2006).

42. Trimmer, C. *et al.* Genetic variation across the human olfactory receptor repertoire alters odor perception. *Proc. Natl. Acad. Sci.* **116**, 9475–9480 (2019).

43. Chesi, A. & Grant, S. F. A. The Genetics of Pediatric Obesity. *Trends Endocrinol. Metab.* **26**, 711–721 (2015).

44. Higgins, M. G. *et al.* GWAS and eQTL analysis identifies a SNP associated with both residual feed intake and GFRA2 expression in beef cattle. *Sci. Rep.* **8**, 14301 (2018).

45. Saito, N. *et al.* Involvement of the olfactory system in the induction of anti-fatigue effects by odorants. *PLOS ONE* **13**, e0195263 (2018).

46. Choquette, A. C. *et al.* Association between olfactory receptor genes, eating behavior traits and adiposity: Results from the Quebec Family Study. *Physiol. Behav.* **105**, 772–776 (2012).
