# Supplementary material for: Human subsistence and signatures of selection on chemosensory genes
Source: Commun Biol. 2023 Jul 3;6:683. doi: 10.1038/s42003-023-05047-y (PMC10317983; doi:10.1038/s42003-023-05047-y)
Supplement: Supplementary file 2 — Reporting Summary [file 42003_2023_5047_MOESM2_ESM.pdf]

nature research

Corresponding author(s): Carrie Veilleux & Amanda Melin

Last updated by author(s): Feb 22, 2023

# Reporting Summary

Nature Research wishes to improve the reproducibility of the work that we publish. This form provides structure for consistency and transparency in reporting. For further information on Nature Research policies, see our Editorial Policies and the Editorial Policy Checklist.

## Statistics

For all statistical analyses, confirm that the following items are present in the figure legend, table legend, main text, or Methods section.

| n/a | Confirmed | |
|---|---|---|
| ☐ | ☒ | The exact sample size (*n*) for each experimental group/condition, given as a discrete number and unit of measurement |
| ☐ | ☒ | A statement on whether measurements were taken from distinct samples or whether the same sample was measured repeatedly |
| ☐ | ☒ | The statistical test(s) used AND whether they are one- or two-sided<br>*Only common tests should be described solely by name; describe more complex techniques in the Methods section.* |
| ☐ | ☒ | A description of all covariates tested |
| ☐ | ☒ | A description of any assumptions or corrections, such as tests of normality and adjustment for multiple comparisons |
| ☐ | ☒ | A full description of the statistical parameters including central tendency (e.g. means) or other basic estimates (e.g. regression coefficient) AND variation (e.g. standard deviation) or associated estimates of uncertainty (e.g. confidence intervals) |
| ☐ | ☒ | For null hypothesis testing, the test statistic (e.g. *F*, *t*, *r*) with confidence intervals, effect sizes, degrees of freedom and *P* value noted<br>*Give P values as exact values whenever suitable.* |
| ☒ | ☐ | For Bayesian analysis, information on the choice of priors and Markov chain Monte Carlo settings |
| ☒ | ☐ | For hierarchical and complex designs, identification of the appropriate level for tests and full reporting of outcomes |
| ☒ | ☐ | Estimates of effect sizes (e.g. Cohen's *d*, Pearson's *r*), indicating how they were calculated |

*Our web collection on statistics for biologists contains articles on many of the points above.*

## Software and code

Policy information about availability of computer code

| | |
|---|---|
| Data collection | Open source code used: Neutral Region Explorer, Picard Tools, GATK (HaplotypeCaller, CombineGVCF, GenotypeGVCF), samtools v1.9, vcftools 4.2, Ensembl Variant Effect Predictor (GrCH37), plink v1.9, ALoFT v.1.0, BEAGLE 5.1<br>Custom code (available on https://github.com/GokcumenLab/data): Human Data Preparation.txt (code for variant calling with GATK, initial filtering of variants for quality control with vcftools) |
| Data analysis | Open source code used: vcftools 4.2, R (packages: ecdf, rworldmap), VCFtoTree, PopART (Population Analysis with Reticulate Trees)<br>Custom code (available on https://github.com/GokcumenLab/data): lof.R (code for Loss of Function analyses), Chemosensation_Subsistence_PBS_R_Code.R (code for PBS analyses) |

For manuscripts utilizing custom algorithms or software that are central to the research but not yet described in published literature, software must be made available to editors and reviewers. We strongly encourage code deposition in a community repository (e.g. GitHub). See the Nature Research guidelines for submitting code & software for further information.

## Data

Policy information about availability of data

All manuscripts must include a data availability statement. This statement should provide the following information, where applicable:

- Accession codes, unique identifiers, or web links for publicly available datasets
- A list of figures that have associated raw data
- A description of any restrictions on data availability

All capture/sequencing and g.vcf data has been deposited in the European Genome-Phenome Archive (accession number pending). All other data, including LOF variants (Supplementary Data S1), target coordinates for MYbaits (Supplementary Data S2), and PBS values for each pair of focal populations are available at https://github.com/GokcumenLab/data. An expanded discussion of LOF variant distribution and evolutionary history for two haplotypes of particular interest,

methodological figures (e.g., quality control filtering), and lists of significant PBS SNPs per geographic region, PheWAS results, and summaries of other functional annotations and their references are provided in the Supplementary Information.

# Field-specific reporting

Please select the one below that is the best fit for your research. If you are not sure, read the appropriate sections before making your selection.

☒ Life sciences ☐ Behavioural & social sciences ☐ Ecological, evolutionary & environmental sciences

For a reference copy of the document with all sections, see nature.com/documents/nr-reporting-summary-flat.pdf

# Life sciences study design

All studies must disclose on these points even when the disclosure is negative.

| | |
|---|---|
| Sample size | We sampled 158 adult individuals, representing ~30 individuals per population across 6 populations. We chose 30 individuals per population because this was the maximum possible size given sample availability and budget limitations. Because the Twa and BaKiga populations were genotyped in a previous study, we selected individuals from those populations based on cross-population admixture levels and known relationship status. We used custom baits to target 840 functional and pseudogenized OR genes, 33 functional or pseudogenized TASR genes, and 69 intergenic regions across each individual. |
| Data exclusions | We excluded data at the individual and site levels. Almost all exclusion criteria were pre-established before exclusion. Data from 25 individuals were excluded because of low coverage (<50% variants called) or consistently high read-depth across all loci that may indicate a bias in the capture step for these individuals. We excluded sites on sex chromosomes, and sites that had low site quality scores (< 30), low genotype quality scores (< 20), low site coverage (called in < 75% of individuals). We also excluded sites from regions with unusually low read-depth across most samples (< 20th percentile), which indicate regions where our capture consistently failed. Additionally, we excluded all variants from 4 genes after data collection (OR4C3, OR4C4P, OR4C5, OR8U1), as we discovered in the literature that these genes are duplicated at a separate location on the chromosome in non-reference genomes. |
| Replication | To ensure that our dataset has a minimal technical bias, we implemented standard quality checks at the capture and sequencing levels. In addition, we conducted a read-depth analysis, and based on this analysis; we excluded samples that did not pass a stringent threshold indicating successful capture and accurate variant calling. We detail our efforts to minimize our biases in Methods and Supplementary Methods. |
| Randomization | Individuals were allocated into groups based on their population of origin and the subsistence strategy practiced by that population. |
| Blinding | As detailed in the manuscript, all sequencing, capture, and library preparations as well as variant calling analyses were conducted in a blinded fashion (i.e., all samples are treated equally without knowledge of their geographic origin or cultural affiliations). Thus, we expect no batch effects or other types of biases in our study. |

# Reporting for specific materials, systems and methods

We require information from authors about some types of materials, experimental systems and methods used in many studies. Here, indicate whether each material, system or method listed is relevant to your study. If you are not sure if a list item applies to your research, read the appropriate section before selecting a response.

## Materials & experimental systems

| n/a | Involved in the study | |
|---|---|---|
| ☒ | ☐ | Antibodies |
| ☒ | ☐ | Eukaryotic cell lines |
| ☒ | ☐ | Palaeontology and archaeology |
| ☒ | ☐ | Animals and other organisms |
| ☐ | ☒ | Human research participants |
| ☒ | ☐ | Clinical data |
| ☒ | ☐ | Dual use research of concern |

## Methods

| n/a | Involved in the study | |
|---|---|---|
| ☒ | ☐ | ChIP-seq |
| ☒ | ☐ | Flow cytometry |
| ☒ | ☐ | MRI-based neuroimaging |

## Human research participants

Policy information about studies involving human research participants

| | |
|---|---|
| Population characteristics | Individuals participating in the study were adults from hunter-gather populations and farming populations in Uganda and the Philippines. |
| Recruitment | Participants were recruited following presentations to and discussions with the communities about the broader project, and with individual informed consent. |

| | |
|---|---|
| Ethics oversight | This study is conducted in collaboration with Makerere University and University of the Philippines, Diliman under formal agreements or memoranda of understanding, and approved by the Research and Ethics Committee, Makerere University Faculty of Medicine (protocol 2009-137), the Uganda National Council for Science and Technology (permit HS 617), the National Commission on Indigenous Peoples (NCIP), and the Department of Environment and Natural Resources (permit 03-2010). Consent to saliva collection (Oragene Saliva Collection Kit; DNA Genotek, Ottawa, Canada) and genetic analysis occurred at three levels—local government units, indigenous elder members of each settlement or community, and adult individuals—and in close coordination with the Batwa Development Programme, a nongovernmental cultural organization, or regional officers of the NCIP (Region 2, Barbara Garcia; Region 13, Villarica Lumancas). Our protocol for informed consent was also approved by the Committee for the Protection of Human Subjects, Dartmouth College (protocol 22410), the Institutional Review Board, University of California, Santa Cruz (protocol HS0801367), and the Institutional Review Board, University of Chicago (protocol 16986A). |

Note that full information on the approval of the study protocol must also be provided in the manuscript.
